# Unveiling the biology of defective viral genomes in vitro and in vivo: implications for gene expression and pathogenesis of coronavirus

**DOI:** 10.1186/s12985-023-02189-7

**Published:** 2023-10-06

**Authors:** Ching-Hung Lin, BoJia Chen, Day-Yu Chao, Feng-Cheng Hsieh, Chun-Chun Yang, Hsuan-Wei Hsu, Hon-Man-Herman Tam, Hung-Yi Wu

**Affiliations:** 1 Graduate Institute of Veterinary Pathobiology, College of Veterinary Medicine, National Chung Hsing University, Taichung, 40227 Taiwan; 2 Doctoral Program in Microbial Genomics, National Chung Hsing University and Academia Sinica, Taichung, 40227 Taiwan; 3 Graduate Institute of Microbiology and Public Health, College of Veterinary Medicine, National Chung Hsing University, Taichung, 40227 Taiwan; 4 Department of Post-Baccalaureate Medicine, College of Medicine, National Chung Hsing University, Taichung, 40227 Taiwan

**Keywords:** Coronavirus, Defective viral genome, Gene expression, Pathogenesis, Coronavirus genome structure

## Abstract

**Background:**

Defective viral genome (DVG) is a truncated version of the full-length virus genome identified in most RNA viruses during infection. The synthesis of DVGs in coronavirus has been suggested; however, the fundamental characteristics of coronavirus DVGs in gene expression and pathogenesis have not been systematically analyzed.

**Methods:**

Nanopore direct RNA sequencing was used to investigate the characteristics of coronavirus DVGs in gene expression including reproducibility, abundance, species and genome structures for bovine coronavirus in cells, and for mouse hepatitis virus (MHV)-A59 (a mouse coronavirus) in cells and in mice. The MHV-A59 full-length genomic cDNAs (~ 31 kilobases) were in vitro constructed to experimentally validate the origin of coronavirus DVG. The synthesis of DVGs was also experimentally identified by RT-PCR followed by sequencing. In addition, the alterations of DVGs in amounts and species under different infection environments and selection pressures including the treatment of antiviral remdesivir and interferon were evaluated based on the banding patterns by RT-PCR.

**Results:**

The results are as follows: (i) the structures of DVGs are with diversity, (ii) DVGs are overall synthesized with moderate (MHV-A59 in cells) to high (BCoV in cells and MHV-A59 in mice) reproducibility under regular infection with the same virus inoculum, (iii) DVGs can be synthesized from the full-length coronavirus genome, (iv) the sequences flanking the recombination point of DVGs are AU-rich and thus may contribute to the recombination events during gene expression, (v) the species and amounts of DVG are altered under different infection environments, and (vi) the biological nature of DVGs between in vitro and in vivo is similar.

**Conclusions:**

The identified biological characteristics of coronavirus DVGs in terms of abundance, reproducibility, and variety extend the current model for coronavirus gene expression. In addition, the biological features of alterations in amounts and species of coronavirus DVGs under different infection environments may assist the coronavirus to adapt to the altered environments for virus fitness and may contribute to the coronavirus pathogenesis. Consequently, the unveiled biological features may assist the community to study the gene expression mechanisms of DVGs and their roles in pathogenesis, contributing to the development of antiviral strategy and public health.

**Supplementary Information:**

The online version contains supplementary material available at 10.1186/s12985-023-02189-7.

## Background

Coronaviruses (CoVs) are in the family *Coronaviridae*, order *Nidovirales* [1, 2]. CoVs can infect humans and animals and thus have led to widespread and costly diseases, such as COVID-19 caused by severe acute respiratory syndrome coronavirus 2 (SARS-CoV-2) [3–6]. CoVs contain the largest known viral RNA genome with the length of ~ 30 kilobases (kb). The genome structure consists of a cap, a 5’ untranslated region (UTR), open reading frames (ORFs), intergenic spaces, a 3’ UTR and a 3’ poly(A) tail. Nonstructural proteins (nsps) are derived from the 5’ two-thirds of the genome which contains two ORFs (ORF1a and ORF1b). The structural and accessory proteins, on the other hand, are encoded from subgenomic mRNAs (sgmRNAs), which are synthesized from the other one-third of the genome during coronavirus transcription [7].

Defective viral genomes (DVGs) is a truncated version of the virus genome and can be found in most RNA viruses during infection [8–10]. Because DVGs have been identified to have effects on tumor cells [11], virus replication [12] and pathogenicity [13], research on DVG has regained attention in recent years. In addition to genomes and sgmRNAs, coronaviruses are also able to synthesize DVGs. Prior to the development of next-generation sequencing (NGS), only 9 coronavirus DVG species from mouse hepatitis viruses (MHVs), bovine coronavirus (BCoV), transmissible gastroenteritis virus (TGEV) and infectious bronchitis virus (IBV) have been experimentally identified [14]. Because these previously identified DVGs contain *cis*-acting elements required for gene expression in their 5’ and 3’ termini, they have been intensively employed as surrogates of the ~ 30 kb full-length genome for studies on coronavirus gene expression [15–21]. With the development of NGS, more coronavirus DVG species have been discovered. However, the basic biological characteristics and thus the biological relevance of DVGs in coronavirus gene expression and pathogenesis remain to be defined.

It has been suggested that in Brome mosaic virus that the AU-rich sequence is a hot spot involved in the recombination and synthesis of a smaller size of viral RNA [22]. Since the coronavirus DVGs have been speculated to be synthesized through a copy-choice template switching recombination process [14], whether coronavirus full-length genome bears the sequence features for potential recombination to synthesize DVGs has not been analyzed.

In the current study, in addition to the well-known coronavirus genomes and sgmRNAs, coronavirus DVGs were comprehensively and experimentally analyzed both in vitro and in vivo by RT-PCR with the assistance of nanopore direct RNA sequencing. Furthermore, the biological features of coronavirus DVGs in terms of the structure, classification, abundance, origin, reproducibility and altered species and amounts under different infection environments were also determined. It is expected that the unveiled characteristic of coronavirus DVGs may provide a database for studies of coronavirus gene expression and pathogenesis and thus assist the coronavirus community to develop antiviral strategy.

## Methods

### Viruses, cells and animals

The plaque-purified Mebus strain of BCoV (GenBank: U00735.2) and MHV-A59 (GenBank: NC_048217.1) were used for the study. BCoV-p95 (GenBank: OP296992.1) is a BCoV variant with an altered genome structure of 106 nucleotide mutations obtained from supernatant of HRT-18 cells persistently infected with BCoV. Human rectal tumor (HRT)-18 cells, mouse L (ML) cells, adenocarcinomic human alveolar basal epithelial (A549) cells and baby hamster kidney (BHK) cells were grown in Dulbecco’s modified Eagle’s medium (DMEM) supplemented with 10% fetal bovine serum (HyClone, UT, USA) at 37 °C with 5% CO_2_. Mice were maintained according to the guidelines established in the “Guide for the Care and Use of Laboratory Animals” prepared by the Committee for the Care and Use of Laboratory Animals of the Institute of Laboratory Animal Resources Commission on Life Sciences, National Research Council, USA. The animal study was reviewed and approved (IACUC No.: 108–110) by the Institutional Animal Care and Use Committee of National Chung Hsing University, Taiwan.

### Nanopore direct RNA sequencing and data analyses

For nanopore direct RNA sequencing, total cellular RNA was collected from BCoV-infected HRT-18 cells and MHV-A59-infected ML cells at a multiplicity of infection (MOI) of 0.1. Total cellular RNA was collected at 24 hours (for BCoV) or 20 hours (for MHV-A59) postinfection. In addition, 3-week-old male and specific pathogen-free BALB/c mice (BioLASCO Taiwan Co., Ltd.) were infected by intraperitoneal inoculation of 10^6^ PFU of MHV-A59 in 500 µl of DMEM and total cellular RNA was harvested from the liver at 3 days postinfection. TRIzol (Thermo Fisher Scientific, Waltham, USA) was used to extract total cellular RNA and 500 ng of poly(A)-containing RNA was used for library preparation according to the manufacturer’s instructions (SQK-RNA001, Oxford Nanopore Technologies). Note that ENO2 mRNA, which was added during the library preparation for nanopore direct RNA sequencing supplied by SQK-RNA001 kit (Oxford Nanopore Technologies), was used as an RNA calibrant strand (RCS) to allow assess the RNA degradation during the library preparation based on the coverage of reads [23, 24]. Two biological replicates were performed for nanopore direct RNA sequencing. The data processing codes for basecalling, alignment, and file transformation and primary alignment filtering were as follows: (i) guppy_basecaller --recursive --flowcell FLO-MIN106 --kit SQK-RNA002 -x cuda:0 --u_substitution 0 -i [Input.fast5] -s [output.fastq] --compress_fastq --disable_pings --num_callers 32 --min_qscore 7, (ii) minimap2 -Y -k 14 -w 1 --splice -g 30000 -G 30000 -F 40000 -N 32 --splice-flank = no --max-chain-skip = 40 -u n --MD -a -t 10 --secondary = no [ref] [query] and (iii) Samtools view [Input.sam] -b -f 0 | samtools -@ 10 | bedtools bamtobed -split > [output.bed]. The raw data were filtered with a quality score cutoff of 7 during base-calling. The reads with average quality score higher than 7 were kept for further analysis, and the low-quality reads were removed in this step. To recover the viral recombination reads for BCoV and MHV-A59, the alignment was processed by the minimap2. Furthermore, the secondary and supplementary reads were removed after alignment. The secondary alignments were the inferior alignments, while the supplementary reads were potentially the chimeric reads. Therefore, only the primary alignment reads were retained for further analysis. During the read classification, the reads were classified in the following order: (i) the number of fragments in the RNA transcripts, (ii) whether they contain 3’ UTR, (iii) whether they contain 5’ UTR and (iv) whether they are TRS-relevant. The detailed classification methods are described in Figures S1 and S2, and the associated figure legends. The BAM files were used for (i) the visualization of 5’ and 3’ terminal sequences of DVGs, (ii) analyses of the structures and amounts of coronavirus transcripts, (iii) analyses of the sequence flanking the recombination points of coronavirus DVGs and (iv) analyses of the reproducibility. For reproducibility, RNA transcript with a read count of ≥ 5 was applied and the reproducibility was measured in reads per kilobase per million mapped sequence reads (RPKM) and determined by Spearman’s correlation coefficient [25].

### Preparation of RNA for biological characterization of noncanonical transcripts

To determine the synthesis of BCoV DVGs, HRT-18 cells were infected with 0.1 MOI of BCoV followed by total cellular RNA collection at 2, 8, 24 and 48 h postinfection. To determine the origin of DVGs, the reverse-genetics system of infectious clone MHV-A59-1000 (icMHV), which is divided into 7 cDNA fragments and developed by Dr. Ralph Baric and colleagues, was used [26]. After assembly of the 7 DNA fragments, the full-length viral RNA was in vitro-transcribed using the T7 mMessage mMachine kit (AM1344, Thermo Fisher Scientific, Waltham, USA) with the assembled full-length cDNA as a template. The in vitro-transcribed full-length viral genome was transfected into BHK-MHVR cells. After 48 h of transfection, supernatant (designated MHVVP0) was collected and total cellular RNA was harvested (designated VP0RNA). Plaque assay was employed to detect the virus titer and 0.1 MOI of MHVVP0 was used to infect fresh BHK-MHVR cells. Total cellular RNA was collected (designated VP1RNA). The virus passage step was repeated until VP2RNA was collected.

To evaluate whether the species and the amounts of DVGs were altered in different cells, HRT-18 cells, BHK cells, ML cells and A549 cells were infected with BCoV or BCoV-p95 at an MOI of 0.1, followed by total cellular RNA collection at 24 h postinfection. To determine whether the species and the amounts of DVGs were altered under antiviral selection pressure, HRT-18 cells were infected with 0.1 MOI of BCoV, and after 1 h of infection, HRT-18 cells were treated with the antiviral remdesivir (GS-5734) at final concentrations of 125, 250, 500 or 1000 nM. After 48 h of treatment with remdesivir, total cellular RNA was collected. To evaluate whether the species and the amounts of MHV-A59 DVGs were altered under IFN β treatment, ML cells in 2 ml of DMEM were treated with IFN β at final concentrations of 10^3^, 10^4^ or 10^5^ U/mL. After 16 h of treatment, IFN β-treated ML cells were infected with 0.1 MOI of MHV-A59 followed by total cellular RNA collection at 16 h postinfection. To experimentally determine the synthesis of DVG in mice, 3-week-old male and specific pathogen-free BALB/c mice (BioLASCO Taiwan Co., Ltd.) were infected with 10^6^ PFU of MHV-A59 in 500 µl of DMEM by intraperitoneal inoculation. The livers of MHV-A59-infected mice were collected at 3 days postinfection, and total cellular RNA was prepared.

### Detection of DVGs by RT-PCR

The collected total cellular RNA from aforementioned procedures was used for cDNA synthesis. For this, 10 µg of collected total cellular RNA were used and reverse transcription (RT) was performed by SuperScript III reverse transcriptase (Thermo Fisher Scientific, Waltham, USA). The resulting cDNA was then used for detection of DVGs by PCR and primers (Table S1) and the resulting mixture was heated to 94 °C for 2 min and subjected to 35 cycles of 30 s at 94 °C, 30 s at 55 °C and 90 s at 72 °C. The same cDNA used for detection of 18 S rRNA, coronavirus genome and sgmRNA was heated to 94 °C for 2 min and subjected to 25 cycles of 30 s at 94 °C, 30 s at 55 °C and 20 s at 72 °C.

## Results

### The classification, structure, abundance and reproducibility of coronavirus DVGs

**Fig. 1:**
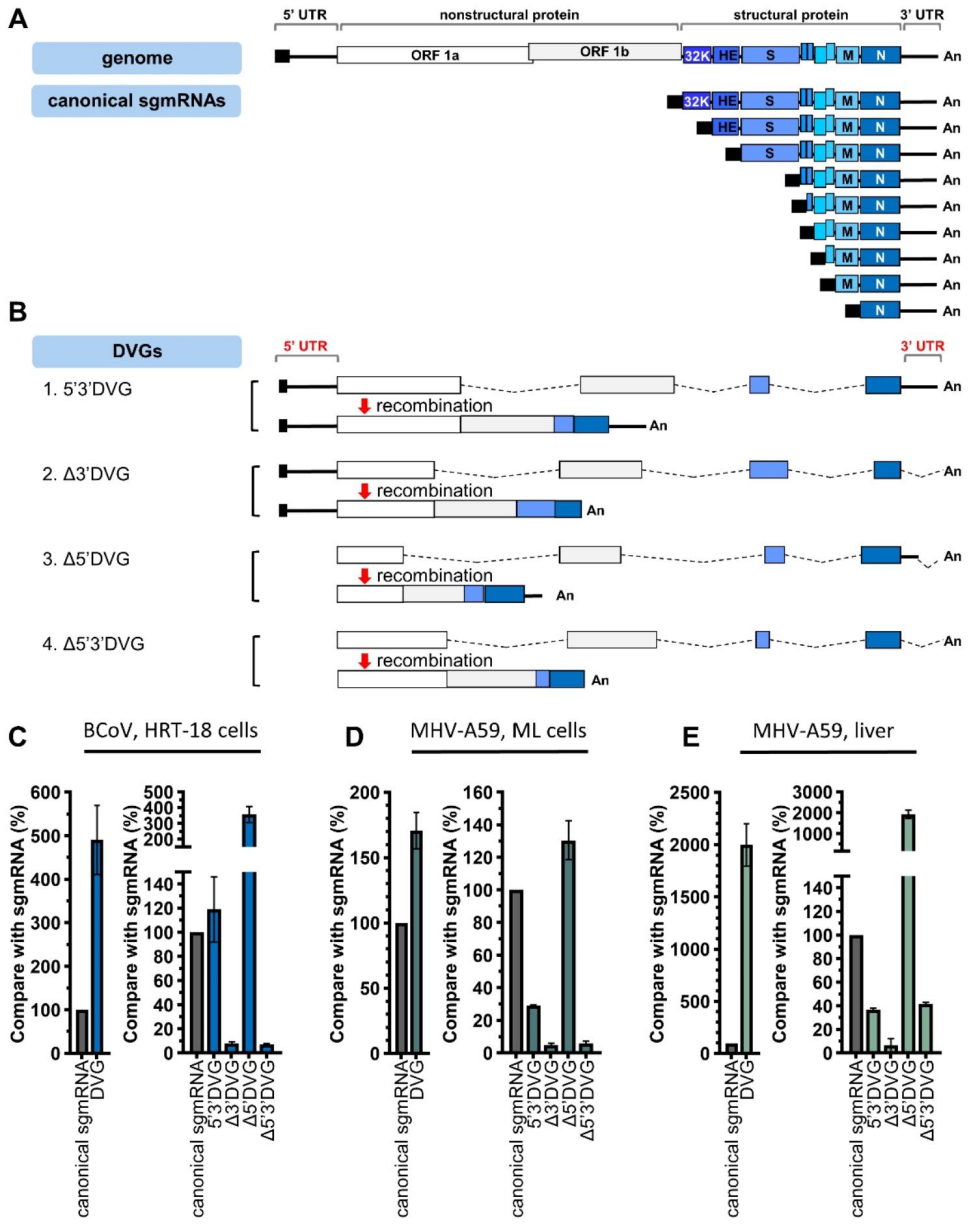
The classification and abundance of coronavirus DVGs based on nanopore direct RNA sequencing. (**A**) The structures of the BCoV full-length genome and canonical sgmRNAs. (**B**) The DVGs can be divided into 4 subgroups Δ5’3’DVG, Δ3’DVG, Δ5’DVG and 5’3’ DVG based on whether DVGs contain 5’ and/or 3’ UTR sequence (partial or complete). Note that DVGs may consist of 1, 2 or more than 2 fragments based on the criteria and definitions for the classes as described in the figure legend of Figure S1. Shown here are DVGs with 4 fragments to emphasize that they are recombination products derived from the different portions of the genome including part of ORF1a, ORF1b, S and N genes. The dashed line indicates the truncated genome in DVGs. (**C**)**-(E)** Left panel: The relative amounts of total DVGs and canonical sgmRNAs in BCoV-and MHV-A59-infected cells and MHV-A59 infected mice. Right panel: The relative amounts of each classified DVG subgroup and canonical sgmRNA in BCoV-and MHV-A59-infected cells and MHV-A59 infected mice. Panels (C)-(E) show the mean of two biological replicates; error bars indicate the standard deviation

RNA transcripts other than those encoded by the coronavirus genome are also synthesized during infection [27]. Based on whether they are relevant to transcription regulatory sequence (TRS) (Figure S1A), a sequence motif from which subgenomic mRNAs (sgmRNAs) are synthesized, coronavirus RNA transcripts are classified into two categories: subgenomic mRNAs (sgmRNAs), which are TRS-relevant transcripts, and DVGs, which are TRS-irrelevant transcripts (Figures S1B and S1C) (Lin et al., unpublished data). The detailed definition for classification of the coronavirus RNA transcripts is illustrated in Figure S1 and explained in the associated figure legend. Based on this classification scheme, DVGs can be divided into 4 subgroups based on whether they contain sequence elements from 3’ UTR or/and 5’ UTR, specifically, DVGs with sequence elements from 3’ UTR and 5’ UTR (5’3’DVG), DVGs with sequence elements from 5’ but not 3’ UTR (Δ3’DVG), DVGs with sequence elements from 3’ but not 5’ UTR (Δ5’DVG) and DVGs without sequence elements from both 5’ UTR and 3’ UTR (Δ5’3’ DVG) (Fig. 1B and S1, and the associated figure legends). In addition, based on the classification defined here and the databases obtained in the current study from nanopore direct RNA sequencing (https://osf.io/cm7z6/) for bovine coronavirus (BCoV) and mouse hepatitis virus (MHV)-A59, the abundances of total DVGs and each subgroup was analyzed. The results suggested that coronavirus DVGs were synthesized abundantly when compared with well-known coronavirus canonical sgmRNAs (Fig. 1 C-1E, left panel). Note that the detailed methods for classification based on the definition of RNA transcripts and the databases obtained from nanopore direct RNA sequencing (https://osf.io/cm7z6/) are shown in Figure S2 and explained in the associated figure legend. The results also suggested that Δ5’DVG is the most abundant subgroup among the 4 DVG subgroups while very little Δ3’DVG was synthesized either in cells or mice (Fig. 1 C-1E, right panel). In conclusion, DVGs are synthesized abundantly; however, the abundances varied among the classified 4 subgroups both in vitro and in vivo.

**Fig. 2:**
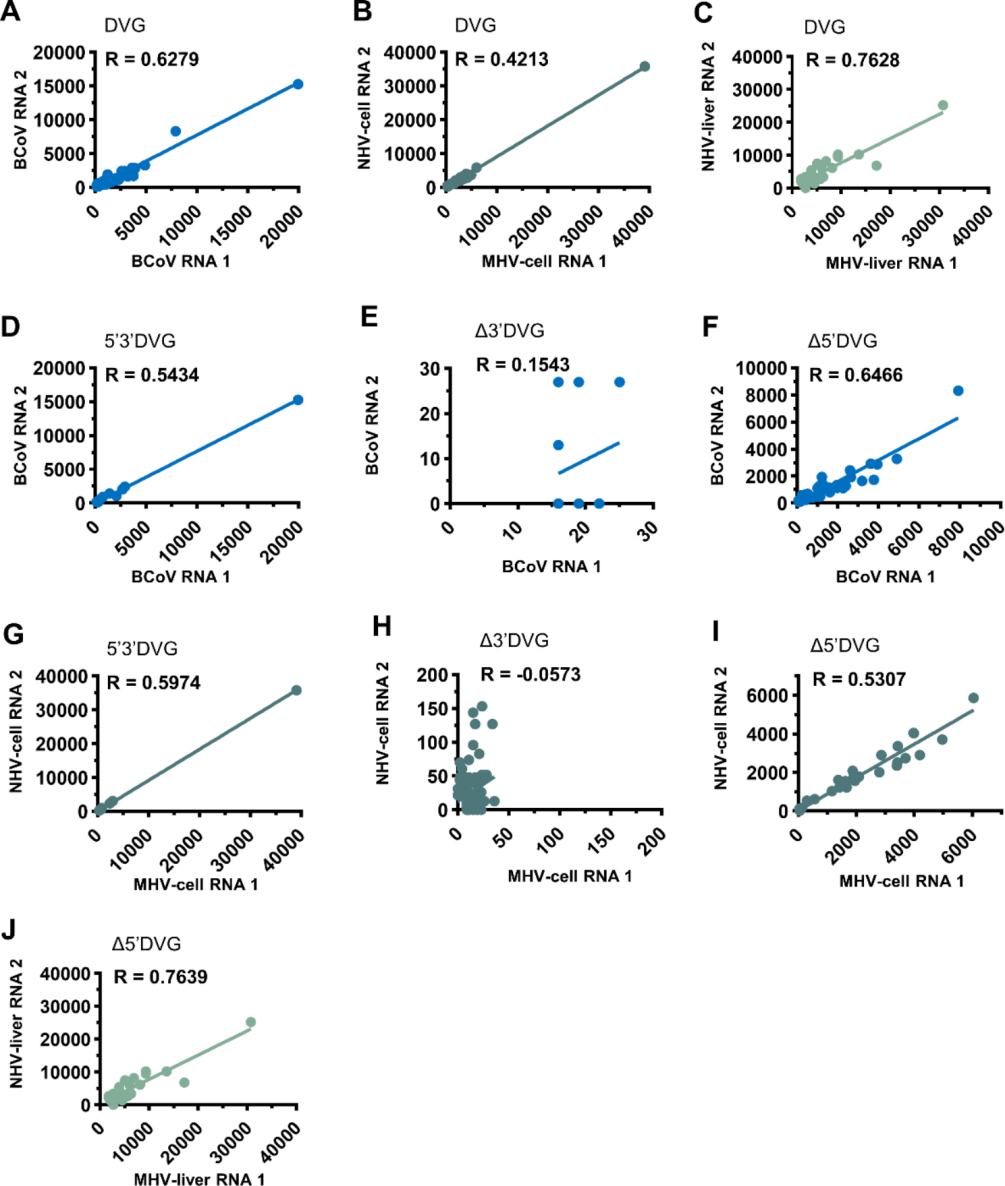
Reproducibility of DVGs. **(A)-(C)** The overall reproducibility of DVGs for BCoV in BCoV-infected cells, MHV-A59-infected cells and mice. **(D)-(F)** The reproducibility of 5’3’DVG, Δ3’DVG and Δ5’DVG in BCoV-infected cells. **(G)-(I)** The reproducibility of 5’3’DVG, Δ3’DVG and Δ5’DVG in MHV-A59-infected cells. **(J)** The reproducibility of Δ5’DVG in MHV-A59-infected mice. Reproducibility was measured for transcripts with a read count of ≥ 5 based on RNA (BCoV RNA1 and BCoV RNA2) collected from the two biological replicates. The reproducibility is measured in reads per kilobase per million mapped sequence reads (RPKM) and evaluated by Spearman’s correlation coefficient. R = 0.000-0.3999, low reproducibility; R = 0.4000-0.5999, moderate reproducibility; R = 0.6000-1.0000, high reproducibility

Whether the DVGs in coronavirus are opportunistically synthesized or reproducible remains unknown. To this end, two independent infection experiments were performed with the same BCoV and MHV-A59 inoculum for nanopore direct RNA sequencing, and transcripts with a read count of ≥ 5 is used for Spearman’s correlation coefficient test to examine the reproducibility. As shown in Fig. 2, analysis based on nanopore direct RNA sequencing data suggested that the overall reproducibility was high for DVGs in BCoV-infected cells (Fig. 2A) and MHV-A59-infected mice (Fig. 2C) but was moderate in MHV-A59-infected cells (Fig. 2B). For the reproducibility of BCoV DVG subgroups in infected cells (Fig. 2D F), 5’3’DVG and Δ5’DVG were synthesized with moderate (5’3’DVG) to high (Δ5’DVG) reproducibility, but Δ3’DVG was synthesized with low reproducibility. For cell cultures infected with MHV-A59, the subgroups 5’3’DVG (Fig. 2G) and Δ5’DVG (Fig. 2I) were synthesized with moderate reproducibility, but Δ3’DVG (Fig. 2H) was synthesized with low reproducibility. For mice infected with MHV-A59, the subgroup Δ5’DVG was synthesized with high reproducibility (Fig. 2H). Note that the reproducibility data for Δ5’3’DVG of BCoV and MHV-A59 in cell culture and that for 5’3’DVG, Δ3’DVG and Δ5’3’DVG in mice are not available because the number of the transcripts with a read count of ≥ 5 is not sufficient for Spearman’s correlation coefficient test. Consequently, the results suggested that the reproducibility varied between DVG subgroups, but, overall, the DVGs are synthesized with moderate to high reproducibility.

### Identification of coronavirus DVGs by RT-PCR followed by sequencing

**Fig. 3:**
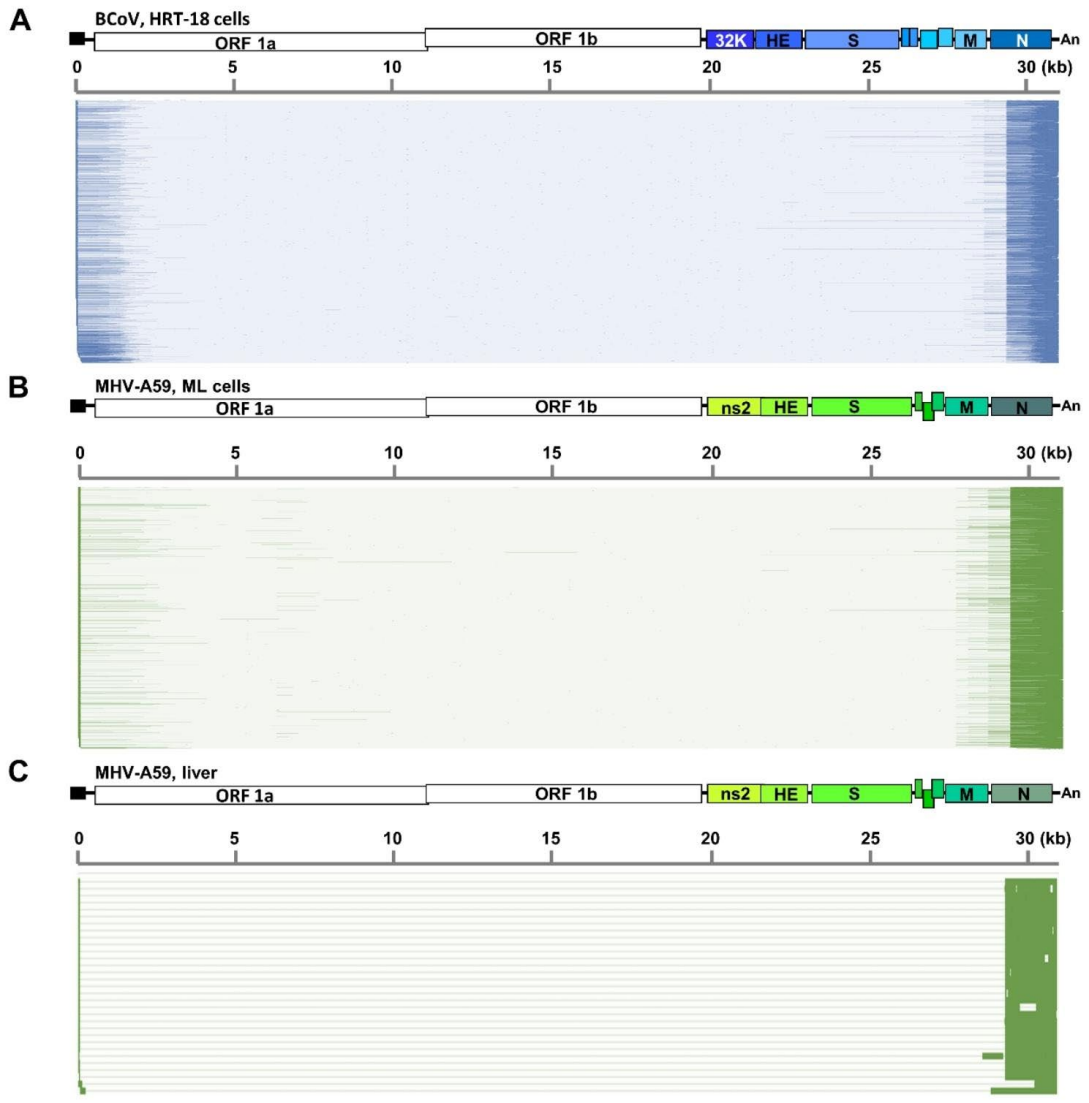
Schematic diagram showing that the 5’3’DVG species contain different lengths of 5’ and 3’ terminal sequences of the genome for BCoV in HRT-18 cells **(A)**, MHV-A59 in ML cells **(B)** and MHV-A59 in mice **(C)**. The 5’ and 3’ terminal sequences are highlighted with darker bars on the x-axis. The results are derived from two biological replicates

**Fig. 4:**
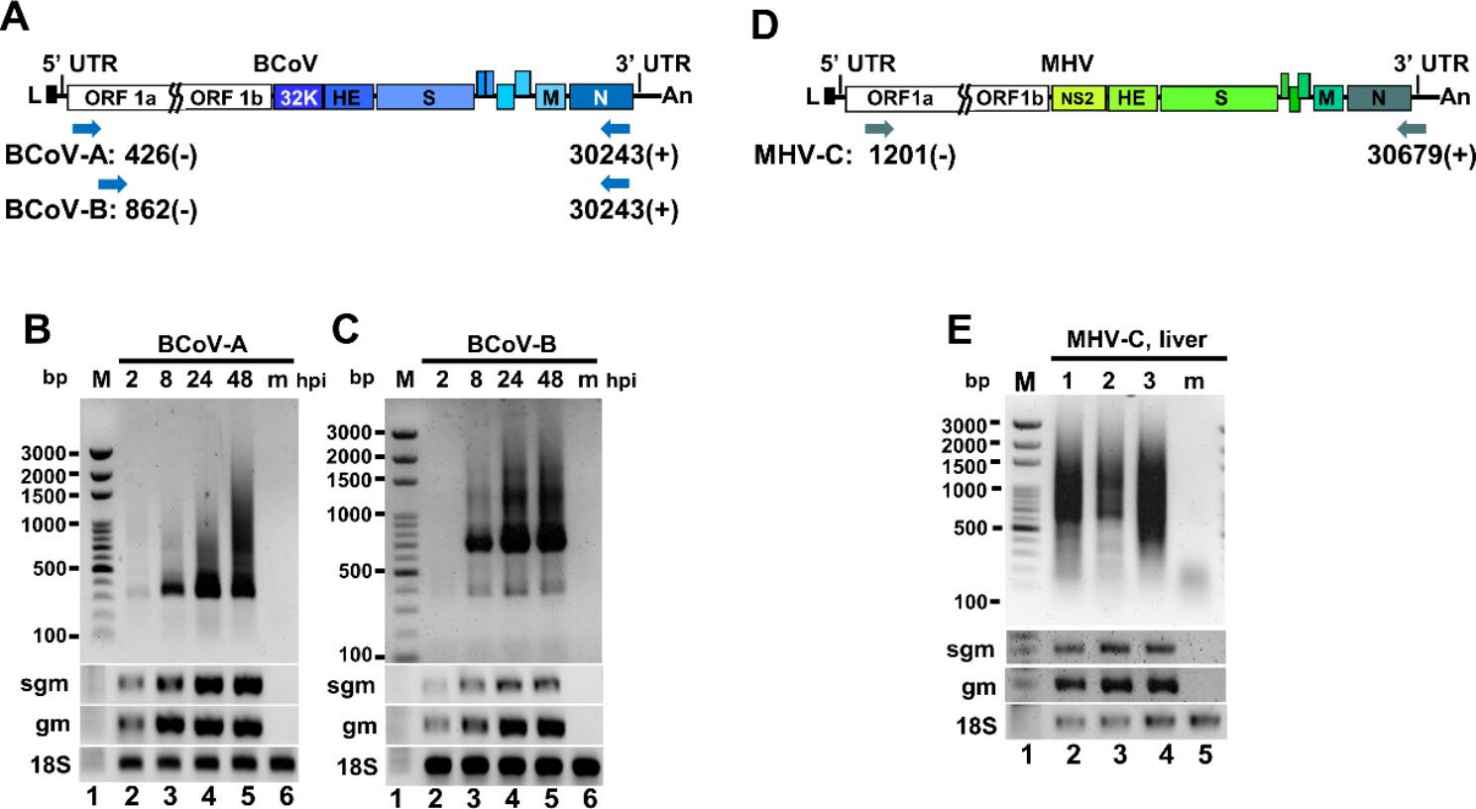
Detection of coronavirus DVGs by RT-PCR. (**A**) Diagram depicting the primer sets used for determining the synthesis of BCoV DVGs in Figures (B)-(C). (**B**)-(**C**) Detection of BCoV DVG synthesis at different time points of BCoV infection by RT-PCR. HRT-18 cells were infected with 0.1 MOI of BCoV followed by total cellular RNA collection at 2, 8, 24 and 48 and RT-PCR with primers shown in Figure (A). (**D**) Diagram depicting the primer sets used for determining the synthesis of MHV-A-59 DVGs in Figure (E). **(E)** Detection of DVGs by RT-PCR (lanes 2–4) from 3 individual mice at 3 days postinfection with MHV-A59. m, mock-infected cells or mice. bp, base-pair; M, DNA size marker; hpi, hours postinfection; sgm, sgmRNA N; gm, genome; 18 S, 18 S rRNA

**Fig. 5:**
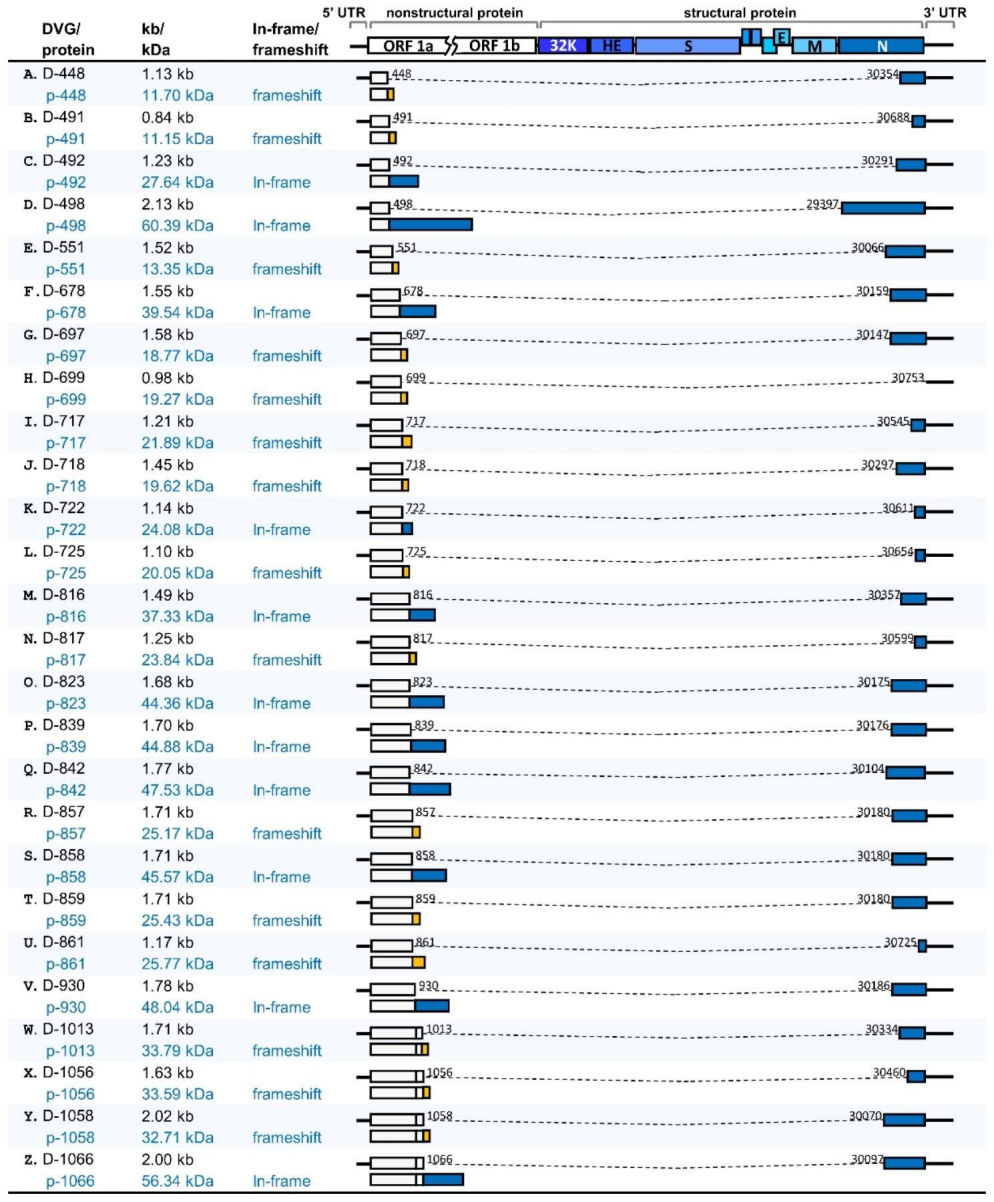
The structures of experimentally identified DVGs and the predicted encoded proteins. The numbers shown in each DVG structure are the nucleotide positions at which the recombination occurs. The dashed line indicates the truncated genome in DVG. The encoded fusion protein from D-448, D-491, D-551, D-697, D-717, D-718, D-725, D-817, D-857 and D-859 contains part of nsp1 and protein resulted from frameshift in N protein gene. The encoded fusion protein from D-492, D-678, D-722, D-816, D-823, D-839, D-842, D858 and D930 contains part of nsp1 and N protein. The encoded fusion protein from D-699 contains part of nsp1 and protein resulted from frameshift in gene from 3’UTR. The encoded fusion protein from D-861 contains part of nsp1 and protein resulted from frameshift in N protein gene and gene from 3’UTR. The encoded fusion protein from D-1013, D-1056 and D-1058 contains nsp1, part of nsp2 and protein resulted from frameshift in N protein gene. The encoded fusion protein from D-1066 contains nsp1 and part of nsp2 and N protein. The encoded fusion protein from D-498 contains part of nsp1 and complete N protein

Based on the data obtained from nanopore RNA direct sequencing, it was suggested that the 5’3’DVG species in BCoV-and MHV-A59-infected cells and MHV-A59 infected mice contained different lengths of 5’ and 3’ terminal sequences of the genome (Fig. 3). Consequently, to experimentally identify the synthesis of DVGs, primers for RT–PCR were designed to bind to the 5’ and 3’ proximal regions of the genome (Fig. 4A). As expected, multiple DVG species were detected by RT–PCR (Fig. 4B), and their amounts increased with the time of infection. The synthesis of DVGs was also identified from the liver of MHV-A59-infected mice (Fig. 4E). With primers which were designed to bind closer to the 5’ and 3’ termini of the genome (BCV81(-), which binds to nucleotide positions of full-length genome from 81 to 110 and BCVEND2(+), which binds to the nucleotide positions of full-length genome from 31,001 to 31,032) followed by sequencing, the 5’3’DVG species (Fig. 5) were also experimentally identified and all of which contain open reading frame(s) (ORFs) from one or different portions of full-length genome.

### DVGs can be synthesized from the full-length coronavirus genome

**Fig. 6:**
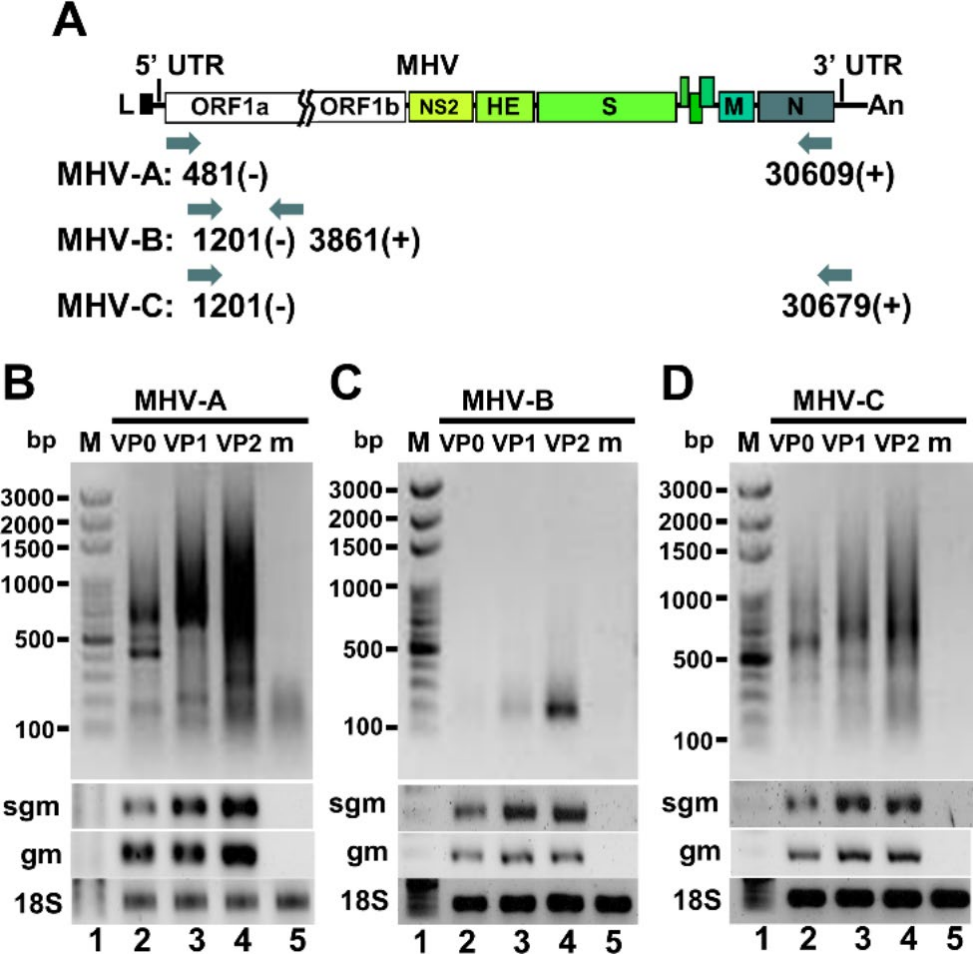
Determination of the origin of DVGs using MHV-A59 full-length genomic cDNA. **(A)** Diagram depicting the primer sets used for determining the synthesis of MHV-A59 DVGs in Figures (B)-(D). **(B)-(D)** Determination of the origin of DVGs using MHV-A59 full-length genomic cDNA. After assembly and in vitro transcription of MHV-A59 full-length genomic cDNA, the full-length viral RNA was transfected into BHK-MHVR cells. After 48 h of transfection, total cellular RNA was harvested (VP0), and the supernatant was collected to infect fresh BHK-MHVR cells. The virus passage step was repeated until VP2. DVGs were detected by RT-PCR with primers shown in Figure (A). bp, base-pair; M, DNA size marker; hpi, hours postinfection; m, mock-infected cells; sgm, sgmRNA N; gm, genome; VP, virus passage; 18 S, 18 S rRNA

To examine whether DVGs can be synthesized from the full-length coronavirus genome, the MHV-A59 full-length genomic cDNAs [26] were employed. In brief, the ~ 31 kb full-genome cDNA, which was divided into 7 DNA fragments, were assembled, transcribed in vitro and transfected into MHVR-BHK cells. Total cellular RNA was collected after 48 h of transfection (designated VP0). The supernatant was also collected from VP0 and then used to infect fresh cells, followed by RNA collection (designated VP1) at 48 hpi. Total cellular RNA at VP2 was similarly prepared, and RT–PCR was performed to detect the synthesis of DVGs. As shown in Fig. 6, with primers binding to different regions of full-length genome (Fig. 6A), multiple DVG species were identified, but with different patterns between passages VP0, VP1 and VP2 (Fig. 6B and D). Because only the assembled full-length genome MHV-A59 was transfected into the cells, these results suggest that the detected DVG species can be synthesized from the transfected full-length coronavirus genome and thus are consistent with the previous studies [27, 28]. In addition, the coronavirus may gradually try to adapt to the new environment by synthesizing different DVG species. This argument is supported by the results in which a new DVG species (Fig. 6C) or different DVG species (Fig. 6B and D) were synthesized during the passages.

### Sequences flanking the recombination points of DVGs are AU-rich

**Fig. 7:**
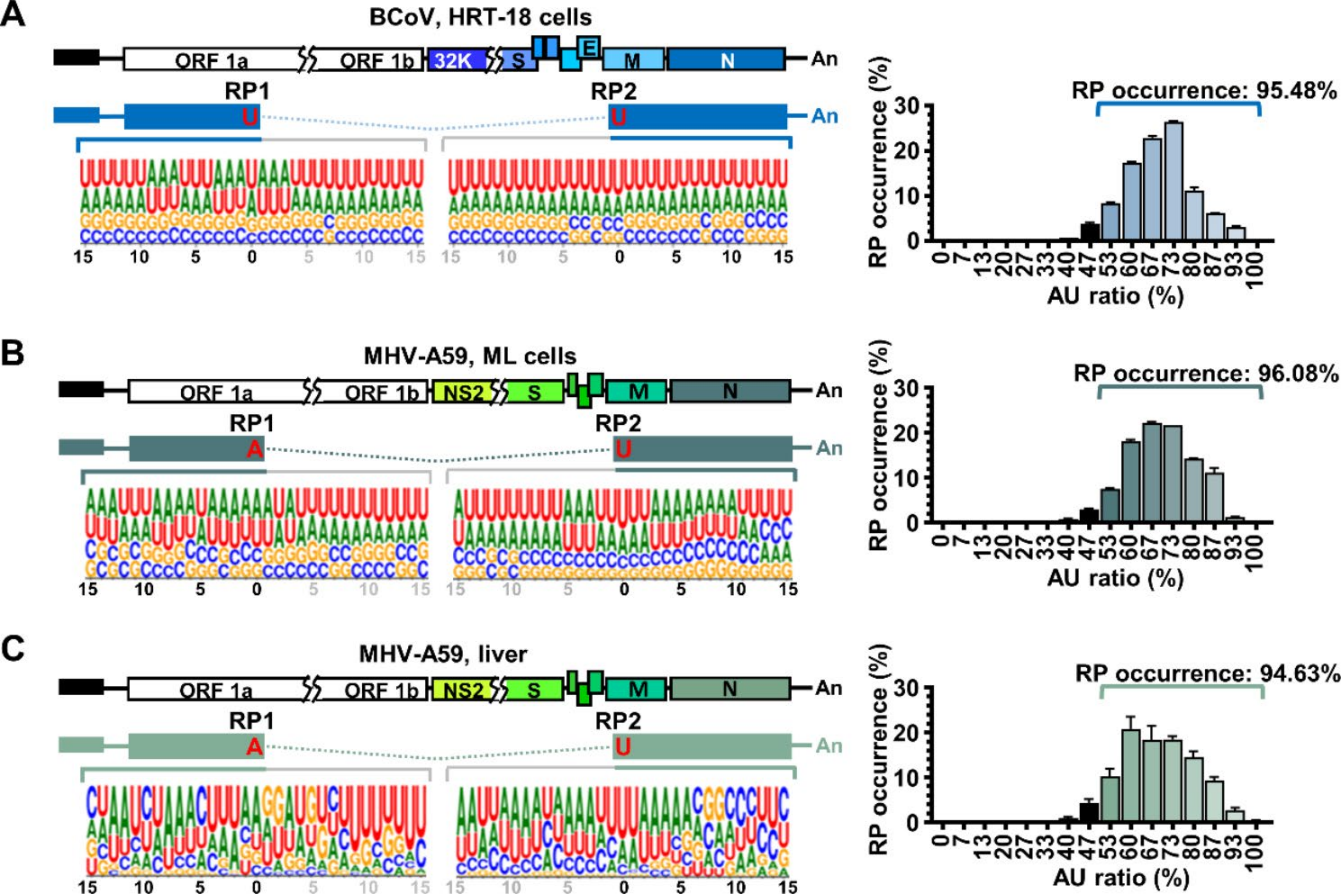
Sequences flanking the recombination points of DVGs are AU-rich. **(A)-(C)** Left panel: Characterization of the sequence flanking the recombination points (RP1 and RP2) of in BCoV-and MHV-A59-infected cells and MHV-A59 infected mice based on databases obtained from nanopore direct RNA sequencing. Right panel: Correspondence between the AU-rich sequence and the occurrence of recombination point. X-axis represents the percentage of AU sequence flanking the recombination points and y-axis represents the percentage of RP occurrence. RP, recombination point. The results are derived from the two biological replicates

Because DVGs are truncated version of the full-length genome and thus it presumably the DVGs are recombination RNA products synthesized by template switching from one end of the nucleotide designated recombination donor point (RDP) to another end of the nucleotide designated recombination acceptor point (RAP), as shown in Fig. 7. In addition, the AU-rich sequence has been demonstrated to be able to facilitate recombination by enhancing virus RdRp to dissociate from one template and “jump” to another template, completing the event of template switching and synthesis of recombinant RNA [22]. To characterize whether the sequence flanking the recombination points bears the feature, sequencing data obtained from the nanopore direct RNA sequence (https://osf.io/cm7z6/) from BCoV-and MHV-A59-infected cells (Fig. 7A and B) and MHV-A59 infected mice (Fig. 7C) were analyzed. It was found that the sequence flanking the recombination point was dominated by an AU-rich sequence (Fig. 7A–C, left panels) and thus the result is consistent with that from previous study [29]. In addition, ~ 95% of the recombination points occurred within the AU-rich sequence, suggesting that the AU-rich sequence (AU ratio more than 50%) flanking the recombination points highly corresponded to the occurrence of recombination (Fig. 7A–C, right panel). Thus, the AU-rich sequence may be one of the factors driving the recombination and thus DVG synthesis.

### The species and amounts of DVGs are altered under different infection environments and selection pressures

As shown in Fig. 2, it is suggested that the DVGs overall are synthesized with moderate to high reproducibility under regular infection with the same virus inoculum. Whether the species and amounts of DVGs synthesized under different infection environments are altered remains unknown. It would be logic to compare the amounts of a specific DVG species by RT-qPCR with a set of specific primer located at upstream and downstream of the recombination point, and then to evaluate whether the species and amounts of DVGs are altered under different infection environments. However, because the genome structures between different DVG species are frequently overlapped and the DVG species may be altered under different infection conditions, it is not likely to design a primer set which can simultaneously identify a specific DVG species synthesized from different infection environments. Furthermore, because PCR tends to amplify cDNA fragments with higher amounts in a complex cDNA mixture, if a specific DVG species cannot be synthesized or can be synthesized but is with lower amounts, other DVG species can instead be detected with the same primer set. Consequently, without the same specific DVG species used for comparison by RT-qPCR, the aim may not be achieved. Due to the aforementioned reasons, the current study was conducted using the same MOI for infection, and coronaviral genome, sgmRNA N and 18 S rRNA were used as controls to compare the synthesis of DVG species under different infection conditions. Based on the banding patterns of detected DVG species with various primer sets, the alterations of DVGs in amounts and species of were evaluated.

**Fig. 8:**
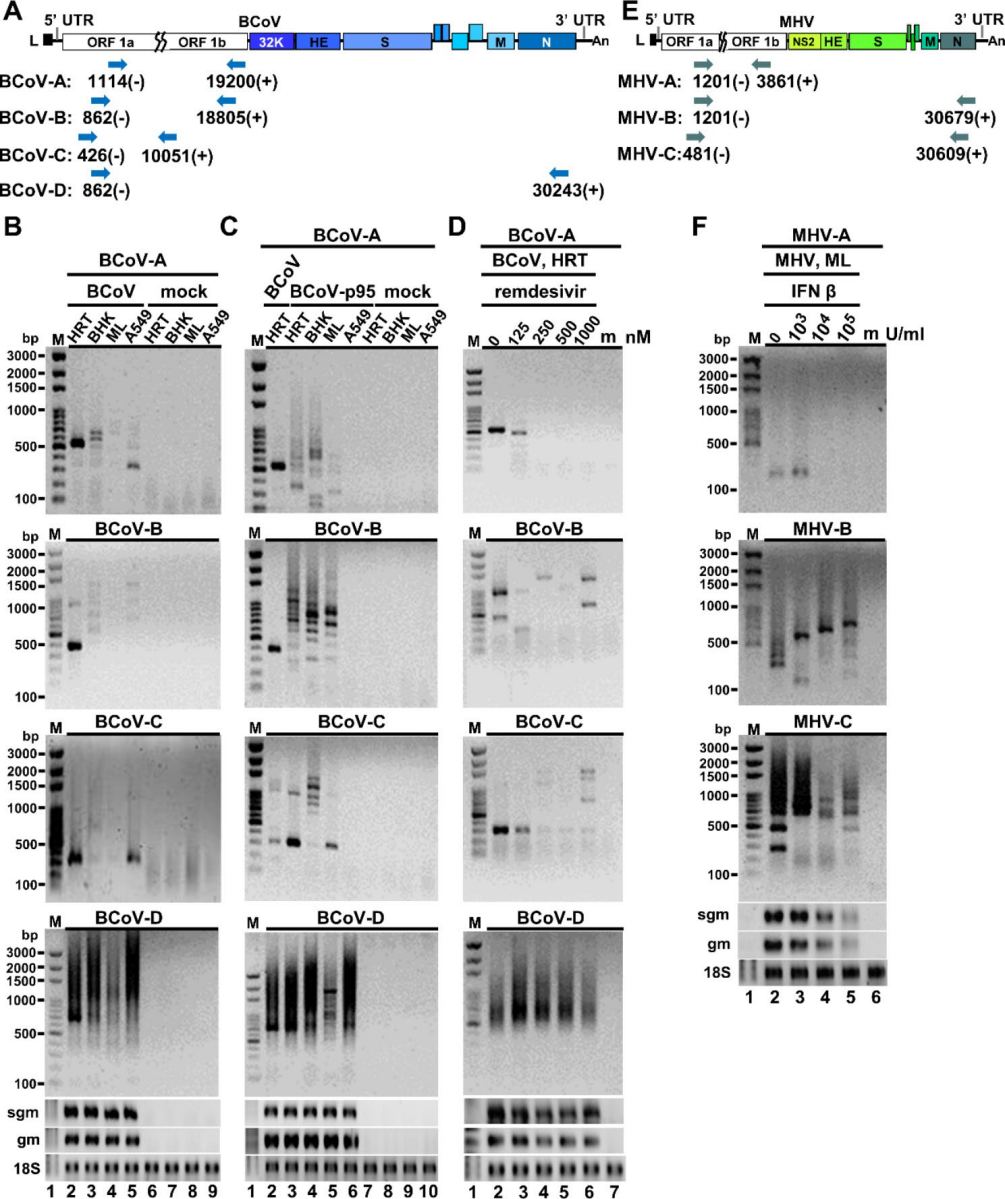
The species and amounts of DVGs are altered under different infection environments and selection pressures. (**A**) Diagram depicting the primer sets used for determining the synthesis of BCoV DVGs in Figures (B)-(D). (**B**) Detection of DVGs synthesized in different cells infected with BCoV by RT-PCR. HRT-18, BHK, ML and A549 cells were infected with BCoV at an MOI of 0.1, followed by total cellular RNA collection at 24 hpi and RT-PCR. The “mock” indicates cells without infection with virus. (**C**) Detection of DVGs synthesized in different cells infected with BCoV-p95 by RT-PCR. HRT-18, BHK, ML and A549 cells were infected with BCoV-p95 at an MOI of 0.1, followed by total cellular RNA collection at 24 hpi and RT-PCR. (**D**) Detection of BCoV DVGs synthesized in HRT-18 cells infected with BCoV (0.1 MOI) and treated with antiviral remdesivir at final concentrations of 125, 250, 500 or 1000 nM. Total cellular RNA was collected at 48 hpi and DVGs were detected by by RT-PCR. (**E**) Diagram depicting the primer sets used for determining the MHV-A59 DVGs in Figure (F). (**F**) Detection of MHV-A59 DVGs synthesized in ML cells infected with MHV-A59 and treated with IFN β by RT-PCR. ML cells in 2 ml of DMEM were treated with IFN β at final concentrations of 10^3^, 10^4^ or 10^5^ U/ml. After 16 h of treatment, IFN β-treated ML cells were infected with 0.1 MOI of MHV-A59 followed by total cellular RNA collection at 16 hpi. bp, base-pair; M, DNA size marker; m, mock-infected cells; HRT, human rectal tumor cells-18; BHK, baby hamster kidney cells; ML, mouse L cells; A549, adenocarcinomic human alveolar basal epithelial cells; hpi, hours postinfection; sgm, sgmRNA N; gm, genome; 18 S, 18 S rRNA

To investigate whether the species and amounts of DVGs synthesized from different cells were altered, HRT-18, BHK, ML and A549 cells were infected with BCoV or BCoV-p95. Note that BCoV-p95 (GenBank: OP296992.1) is a BCoV variant with an altered genome structure of 106 nucleotide mutations obtained from supernatant of HRT-18 cells persistently infected with BCoV. Because a substantial amount of 5’3’DVG species (Fig. 1 C-1D) are with different lengths of 5’ and 3’ proximal sequences of the genome (Fig. 3), primers BCoV-D, which bind to both 5’ and 3’ terminal sequences, were used and multiple DVG species were detected, suggesting DVGs can be synthesized from these cell lines (Fig. 8B). In addition, because not many DVG species are with the sequences which are located distantly from the 5’ and 3’ terminus of full-length genome, with one of the primers binding to the sites distantly from the terminal sequence of full-length genome (BCoV-A: 19,200 (+); BCoV-B: 18,805(+); BCoV-C: 10,051(+), Fig. 8A), fewer DVG species may be detected and this feature allowed us to examine whether the species and amounts of DVGs are altered. Consequently, based on the banding patterns, when compared with the DVG species detected from BCoV-infected HRT-18 cells (Fig. 8B, lane 2), the detected DVG species from BHK, ML and A549 cells (Fig. 8B, lanes 3–5) were either different (with primers BCoV-A and BCoV-B) or the same but with dramatically decreased amounts (with primer BCoV-C), suggesting that the species and amounts of DVGs synthesized from different cell lines infected with the same coronavirus BCoV vary. Similar results were also obtained when the BCoV variant BCoVp95 (Fig. 8C) was used for infection in different cells. The results therefore suggest that the species and amounts of DVGs are altered under different host cells.

To further examine whether such alterations also occurred under selection pressures, BCoV-infected HRT-18 cells were treated with the antiviral drug remdesivir. As shown in Fig. 8D, with the increased amounts of remdesivir, synthesized DVG species and their amounts were also different in comparison with those without treatment of remdesivir, although they all synthesized DVGs (Fig. 8D, primer BCoV-D). The alterations of DVG synthesis also occurred when ML cells were treated with different units of IFN β and infected with MHV-A59, as shown in Fig. 8F. Because DVGs are with the feature of deletion (in comparison with full genome) within its genome structure, RT-PCR product cannot be obtained if one of the primers cannot bind to the DVG. This may explain why different patterns of RT-PCR products were obtained when different sets of primers were applied for samples with the same cDNA or with different treatments shown in Fig. 8. In conclusion, these results together suggest that the species and amounts of DVGs are altered under different infection environments and selection pressures, and thus may play important roles in coronavirus pathogenesis.

## Discussion

It is presumably that the coronavirus DVGs are synthesized through copy-choice template switching mechanism [14]; however, the factors affecting the synthesis remain unclear. In the current study, it is suggested that DVGs can be derived from full-length genome (Fig. 6) and the sequences flanking the recombination point of DVGs are AU-rich. This structural features in coronavirus are consistent with those identified in other RNA viruses in which the AU-rich sequences are associated with the synthesis of DVGs [22]. In addition, the previous study also suggests that the secondary structures near the recombination point as well as the protein factors also play important roles in facilitating recombination events [30] and thus the synthesis of DVGs. In line with this, such a recombination event may also occur with a longer length of DVG as a template, leading to the synthesis of DVG with a shorter length. Consequently, this may increase the diversity of DVG species and possibly the protein species, contributing to coronavirus pathogenesis. Consequently, it is important to determine the synthesis mechanism of coronavirus DVGs. The identified structural features including AU-rich sequences and secondary structures as well as the proteins involved are all potential antiviral targets, contributing to disease control.

There are various definitions and classifications regarding the coronavirus RNA transcripts. The differences in definition and classification between the current study and others [28, 31] are clarified as follows. Non-canonical subgenomic RNAs (nc-sgRNAs) defined by Nomburg et al., [31] suggest that nc-sgRNAs are deleted versions of coronaviral genome with recombination points and are not associated with TRS. Based on the definition, nc-sgRNAs belong to the DVGs (Δ5’3’DVG, Δ3’DVG, Δ5’DVG and 5’3’ DVG) with two or more than two fragments, but not the noncanonical sgmRNAs, in the current study based on the classification criteria of RNA transcripts illustrated in Figures S1 and S2, and the associated figure legends. Note that the noncanonical sgmRNAs defined in the current study are associated with TRS (Figures S1 and S2). On the other hand, the defective interfering (DI) RNAs in DI particles defined by Girgis et al., [28] are coronavirus RNA transcripts which maintain the ability to replicate and can be packaged. Because the defined DI RNA can replicate, they must contain the essential 5’ and 3’ UTR sequences derived from genome for replication. Thus, since the DI RNAs contain 5’ and 3’ UTR sequences and they are not associated with TRS, they belong to 5’3’DVG based on the classification criteria of DVGs in the current study (Figures S1 and S2).

It has been suggested that the DVGs in Sendai virus can stimulate innate immunity [32]. It remains unclear whether coronavirus DVGs bear the structures related to the stimulation of innate immunity. For example, it remains to be determined that whether all of the coronavirus DVG species have the structure of 5’ cap. If the coronavirus DVGs have no cap but bear 5′ triphosphate, during DVG synthesis, the DVGs with 5′ triphosphate may stimulate innate immunity and thus may affect the pathogenesis. On the other hand, if the coronavirus DVGs have cap structure, DVGs may have potential to encode proteins because the identified DVG species contain ORF(s) from one or different portions of full-length genome based on the results in Fig. 5 and obtained from nanopore RNA direct sequencing. Alternatively, it is also possible that some of the DVG species bear the cap structure, but others do not. In either case, such diverse structural features may play important roles in coronavirus pathogenesis. It is worthy of note that, because there are too many DVG species in infected cells, the read number for each DVG species is not high although collectively the total amount of DVGs are abundant and higher than that of canonical sgmRNA (Fig. 1 C-1E). Consequently, it is proposed that DVGs may exert their function in populations but not in individuals either by their structures or by their encoded proteins. Thus, understanding the biological characteristics of DVGs in the current study is also a critical step to explore the mechanism of coronavirus pathogenesis.

Based on the results above, DVG species and their amounts are altered under different infection conditions. Such alteration may be a way for coronavirus to respond to environmental changes and may also contribute to coronavirus pathogenesis. This argument may be one of the reasons why infection of different cells or organs with the same coronavirus leads to different pathologic outcomes. It is speculated that the alteration in DVG species and amounts may suggest the existence of a related regulatory structure or molecule. Alternatively, it is also likely that the alteration may be caused by stochastic variation in different environments. However, the mechanisms of how DVG species and their amounts are altered in response to the different infection conditions remain unclear and thus need to be elucidated. Furthermore, since (i) the synthesized DVG species may differ depending on the infection environments (Fig. 8) and (ii) some of the coronavirus DVGs can replicate and can be packaged into virus particles [15, 28], the DVG species in the new host cells could be from the last passage of the host cells or newly synthesized from the new cells. Consequently, the DVG species in virus particles transmitted among different hosts may also be different and may lead to different effects on infection. Lastly, the selected DVG population may potentially assist coronavirus in developing resistance against the same pressure, posing a concern in controlling coronavirus diseases.

## Conclusions

With the assistance of nanopore RNA direct sequencing, we in the current study experimentally revealed the fundamental characteristics of coronavirus DVGs both in vitro and in vivo. The biological features of coronavirus DVGs in terms of abundance, reproducibility, and variety extend the current model for coronavirus gene expression. The unveiled characteristics of coronavirus DVGs in terms of abundance, reproducibility, the variety of the DVG structures and their protein-coding potential may contribute to the pathogenesis. In addition, the findings that the amounts and DVG species are alterted under different infection environments and selection pressures may further contribute to virus fitness and thus the pathogenesis. Consequently, the current study may contribute to a variety of biomedical studies including the synthesis mechanism of DVGs and its role in pathogenesis, contributing to development of antiviral strategy.

### Electronic supplementary material

Below is the link to the electronic supplementary material.

Supplementary Material 1

## Data Availability

Code for the analyses described in this study is available at https://github.com/BJ-Chen-Eric/The-biology-of-coronavirus-noncanonical-transcripts-in-vitro-and-in-2-vivo/tree/main. The sequencing data are deposited into the Open Science Framework (OSF) at https://osf.io/cm7z6/.

## Abbreviations

CoV: Coronavirus
SARS-CoV-2: Severe acute respiratory syndrome coronavirus 2
UTR: Untranslated region
NGS: Next-generation sequencing
ORF: Open reading frame
Nsp: Nonstructural protein
sgmRNA: Subgenomic mRNA
IBV: Infectious bronchitis virus
TGEV: Transmissible gastroenteritis virus
DVG: Defective viral genome
MHV: Mouse hepatitis viruses
BCoV: Bovine coronavirus
BHK cells: Baby hamster kidney cells
HRT-18 cells: Human rectal tumor-18 cells
ML cells: Mouse L cells
A549 cells: Adenocarcinomic human alveolar basal epithelial cells
MOI: Multiplicity of infection
RPKM: Reads per kilobase per million mapped sequence reads
VP: Virus passage
icMHV: Infectious clone MHV-A59-1000
