# Supplementary material for: Unveiling the biology of defective viral genomes in vitro and in vivo: implications for gene expression and pathogenesis of coronavirus
Source: Virol J. 2023 Oct 6;20:225. doi: 10.1186/s12985-023-02189-7 (PMC10559480; doi:10.1186/s12985-023-02189-7)
Supplement: Supplementary file 1 — Supplementary Material 1 [file 12985_2023_2189_MOESM1_ESM.docx]

**Supplementary data**

**Unveiling the biology of defective viral genomes *in vitro* and *in vivo*: implications for gene expression and pathogenesis of coronavirus**

Ching-Hung Lin^1^, BoJia Chen^2^, Day-Yu Chao^2^, Feng-Cheng Hsieh^1^, Chun-Chun Yang^1^, Hsuan-Wei Hsu^1^, Hon-Man-Herman Tam^1^, Hung-Yi Wu^1*^

**1** Graduate Institute of Veterinary Pathobiology, College of Veterinary Medicine, National Chung Hsing University, Taichung 40227, Taiwan

**2** Graduate Institute of Microbiology and Public Health, College of Veterinary Medicine, National Chung Hsing University, Taichung 40227, Taiwan

*Corresponding Author


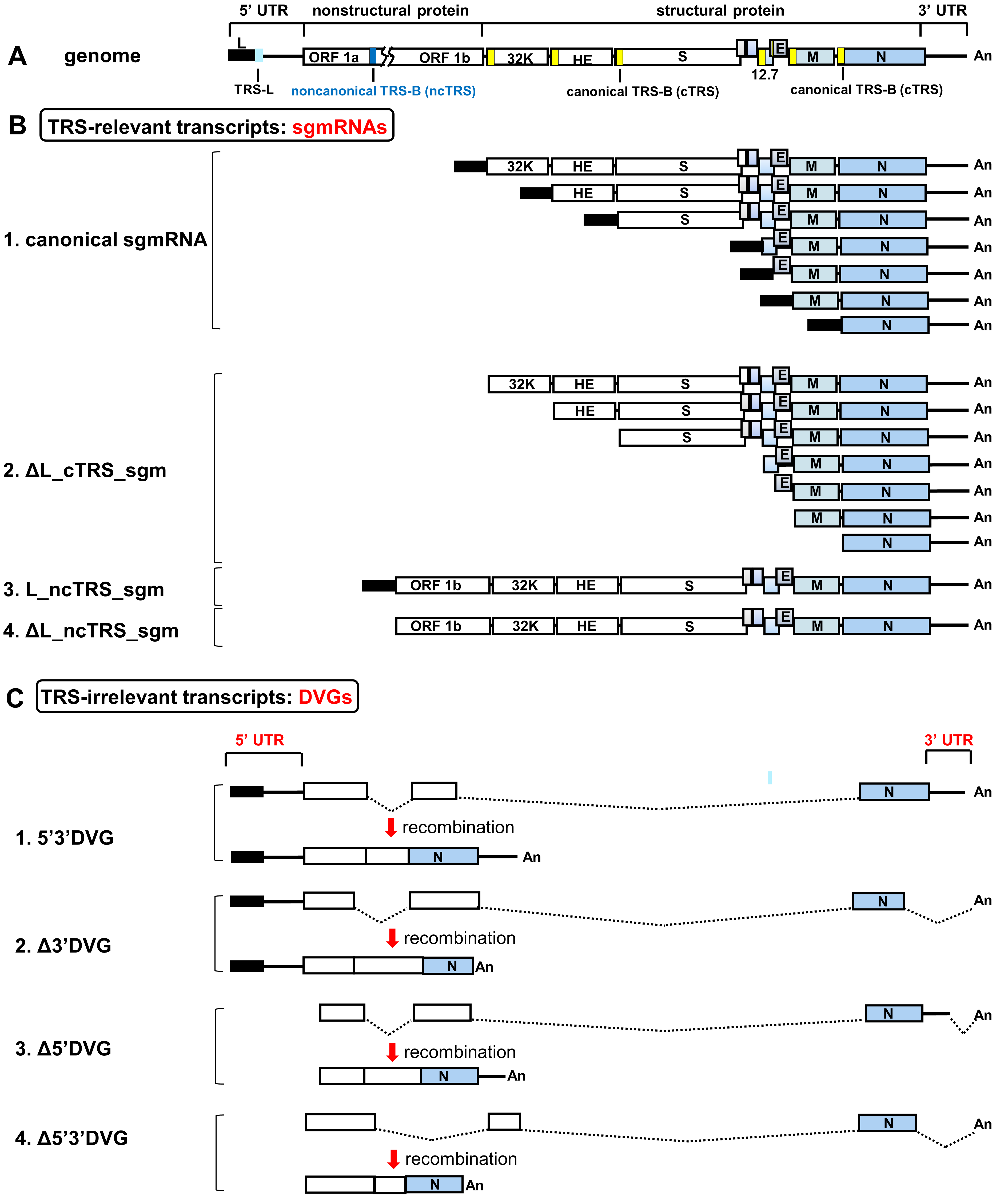


**Figure S1. The classification of coronavirus RNA transcripts. (A)** The genome structure of BCoV. TRS-L, noncanonical TRS-B (ncTRS) and canonical TRS-B (cTRS) are denoted with solid light blue rectangle, solid dark blue and solid yellow rectangle, respectively. The ncTRS shown here is an example located in ORF1a gene, but ncTRSs can also occur in other positions of the genome. L, leader. **(B)** Schematic diagram showing the structures of BCoV TRS-relevant transcripts (sgmRNAs). The TRSs used for synthesis of the main structural and assessor sgmRNAs are defined as canonical TRSs (cTRSs) (solid yellow rectangle, Figure S1A) and thus the synthesized sgmRNAs with leader sequence are defined as canonical sgmRNAs (1. canonical sgmRNA, Figure S1B). If sgmRNAs have no leader sequence, but have a 5’ sequence derived from the sequence positioned within the 50 nucleotides of cTRS, they are defined as leaderless canonical sgmRNAs (2. ΔL_cTRS_sgm, Figure S1B). Consequently, other TRSs, which are not used for synthesis of the main structural and assessor sgmRNAs, are defined as noncanonical TRSs (ncTRSs) (solid dark blue rectangle, Figure S1A), and sgmRNAs synthesized from these sites with leader sequence are then defined as noncanonical sgmRNAs (3. L_ncTRS_sgm Figure S1B). Similarly, if sgmRNAs have no leader sequence, but have a 5’ sequence derived from the sequence positioned within the 50 nucleotides of ncTRS, they are defined as leaderless noncanonical sgmRNAs (4. ΔL_ncTRS_sgm, Figure S1B). **(C)** Schematic diagram showing the structures of BCoV TRS-irrelevant transcripts (DVGs). Because coronavirus is prone to recombination, the synthesized RNA transcripts may contain one or more than one fragment of sequence derived from the coronavirus full-length genome (the dashed line indicates the deleted genome in DVG). Consequently, the truncated versions of the TRS-irrelevant transcripts that are (i) derived from the recombination and (ii) TRS-irrelevant are defined as DVGs. Finally, based on whether the DVGs contain sequence elements of 3’ UTR and/or 5’ UTR (partial or complete), the DVGs are further classified as 5’3’DVG, DVG with sequence elements from 3’ UTR and 5’ UTR; Δ5’DVG, DVG with a sequence element from 3’ UTR but not 5’ UTR; Δ3’DVG, DVG with a sequence element from 5’ UTR but not 3’ UTR; Δ5’3’DVG, DVG lacking sequence elements from 3’ UTR and 5’ UTR. UTR, untranslated region; ORF, open reading frame; An, poly(A) tail; 32K, 32 kDa protein; HE, hemagglutinin/esterase; S, spike protein; 12.7, 12.7 kDa protein; E, envelope protein; M, membrane protein; N, nucleocapsid protein; DVG, defective viral genome; sgm, subgenomic mRNA; L, leader; c, canonical; nc, noncanonical; TRS, transcription regulatory sequence.


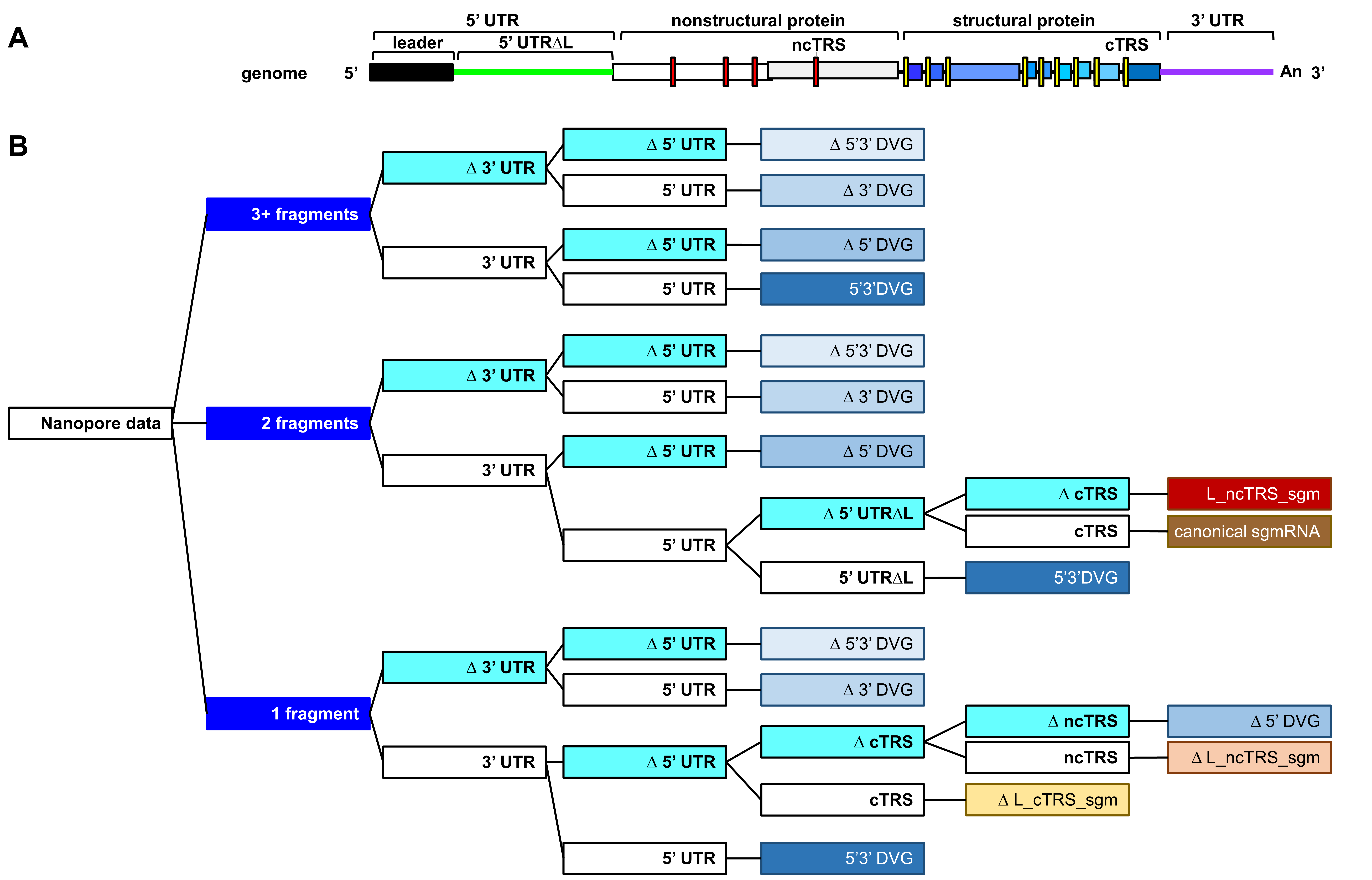


**Figure S2. The detailed classification methods based on the databases obtained from nanopore direct RNA sequencing. (A)** The coronavirus genome structure. L, leader; 5’ UTRΔL, 5’ UTR without leader sequence; ncTRS, body noncanonical TRS; cTRS, body canonical TRS. **(B)** The flow chart depicting the classification methods for coronavirus RNA transcripts. Based on the definitions and classification of coronavirus transcripts described in Figure S1 and the results obtained from nanopore direct RNA sequencing, the criteria and the methods for classification of RNA transcripts are explained as follows. For TRS-relevant transcripts (that is, sgmRNAs, Figure S1B), based on the coronavirus sgmRNA synthesis mechanism, leader-containing sgmRNAs (canonical sgmRNA and L_ncTRS_sgm, Figure S1B) contain (i) a leader sequence derived from the cTRS or ncTRS and (ii) a genome body which contains part of genome followed by a complete 3’UTR and poly(A) tail, and thus contains two fragments of sequences derived from the full-length genome. The leaderless sgmRNAs (ΔL_cTRS_sgm and ΔL_ncTRS_sgm, Figure S1B) have no leader sequence, but have a 5’ sequence derived from the sequence positioned within the 50 nucleotides of the cTRS or ncTRS located at the genome body followed by a complete 3’UTR and poly(A) tail, and therefore contain only one portion from the full-length genome. For TRS-irrelevant transcripts (that is, DVGs, Figure S1C), because coronavirus is prone to recombination, the DVGs may contain one or more than one fragment of sequence derived from the coronavirus full-length genome. Based on the criteria described above, the databases of coronavirus RNA transcripts obtained from nanopore direct RNA sequencing are classified in the following order: (i) the number of fragments in the RNA transcripts, (ii) whether they contain a 3’ UTR, (iii) whether they contain a 5’ UTR and (iv) whether they are TRS-relevant. For DVG classification, including Δ5’DVG, based on (i) the definition of DVGs (Figure S1), (ii) the classification method explained above and (iii) whether the DVGs contain 3’ UTR and/or 5’ UTR sequence elements (partial or complete), the DVGs are further classified into Δ5’3’DVG, Δ3’DVG, Δ5’DVG and 5’3’ DVG. Regarding the classification of Δ5’DVG, because DVGs with two (2 fragments) or more than two fragments (3+ fragments) (i) are recombination products (Figure S1C), (ii) are TRS-irrelevant and (iii) lack sequence elements derived from 5’ UTR, they are therefore classified as Δ5’DVGs according to the definition above. For DVGs with only one fragment (1 fragment), if DVG species are without any sequence derived from the 5’ UTR and with a partial 3’ UTR, they are then classified as Δ5’DVGs because they contain a partial 3’ UTR sequence followed by a poly(A) tail and thus are recombination products based on the definition above. On the other hand, if DVG species (1 fragment) have an intact 3’ UTR sequence followed by a poly(A) tail, they have potential to be degradation products from the 5’ termini, and thus are not classified as DVGs and thus do not fall into the Δ5’DVG category as defined.


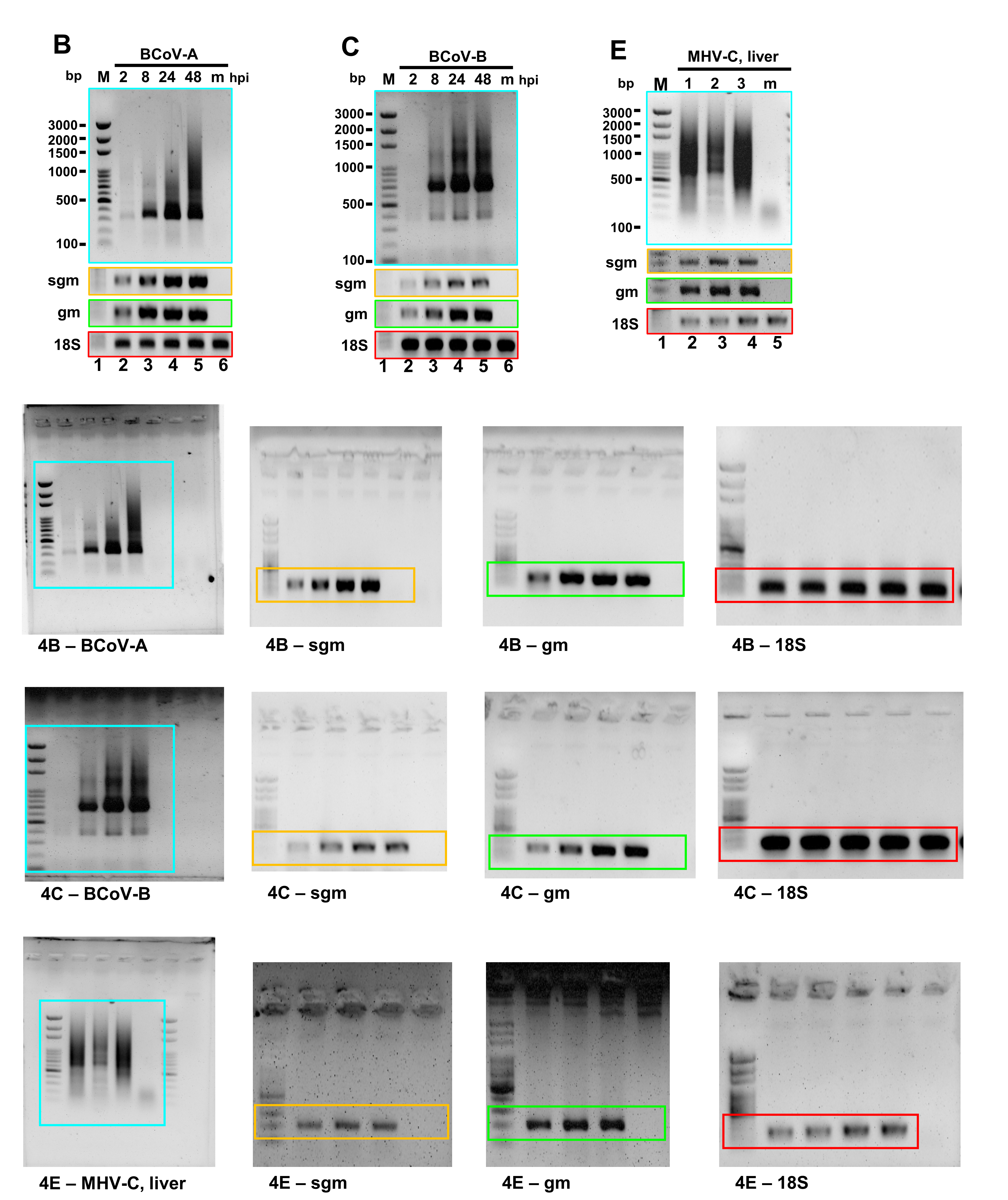


**Figure S3. Uncropped gels for Figures 4B, 4C and 4E.**


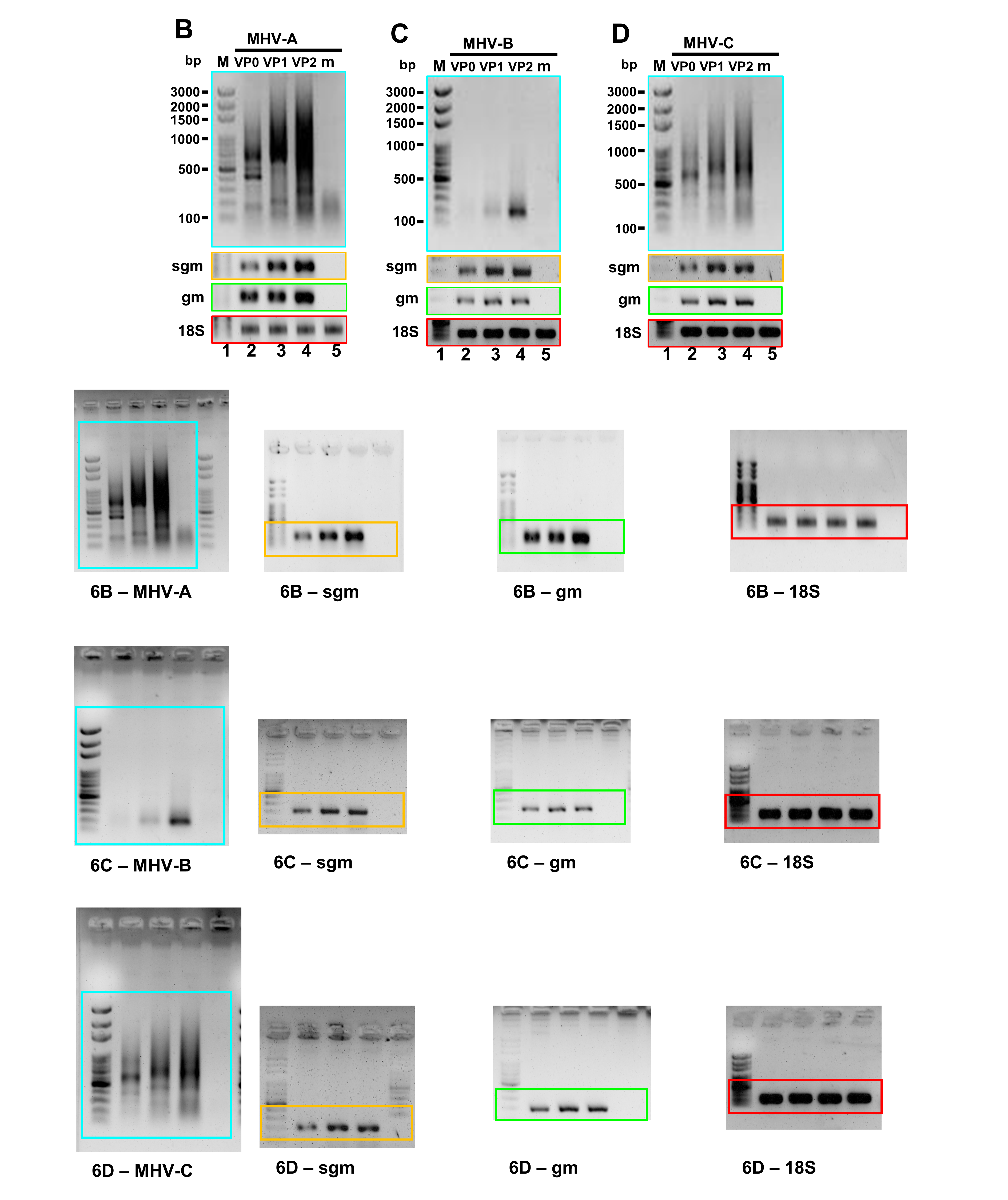


**Figure S4. Uncropped gels for Figures 6B, 6C and 6D.**


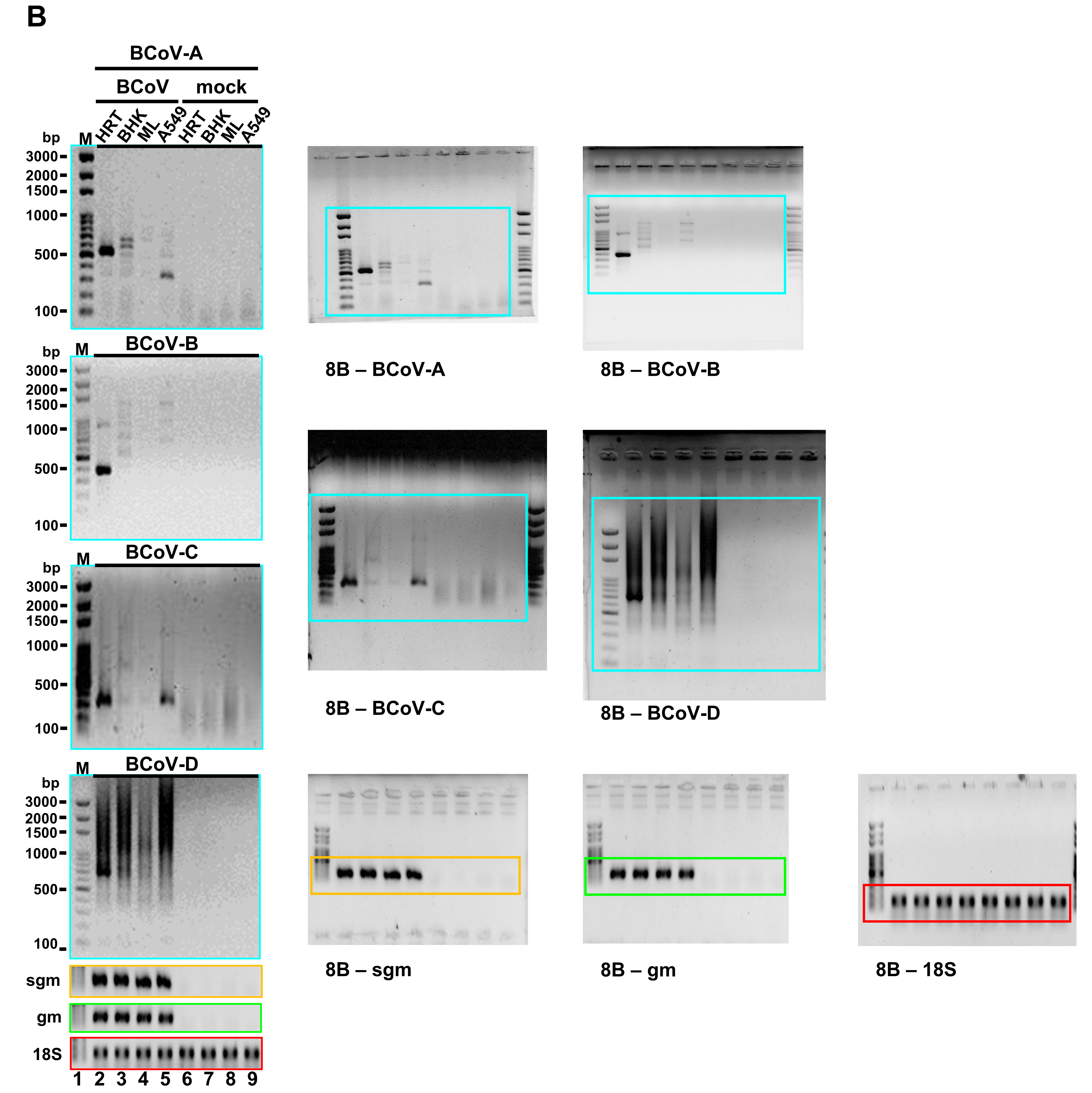


**Figure S5. Uncropped gels for Figure 8B.**


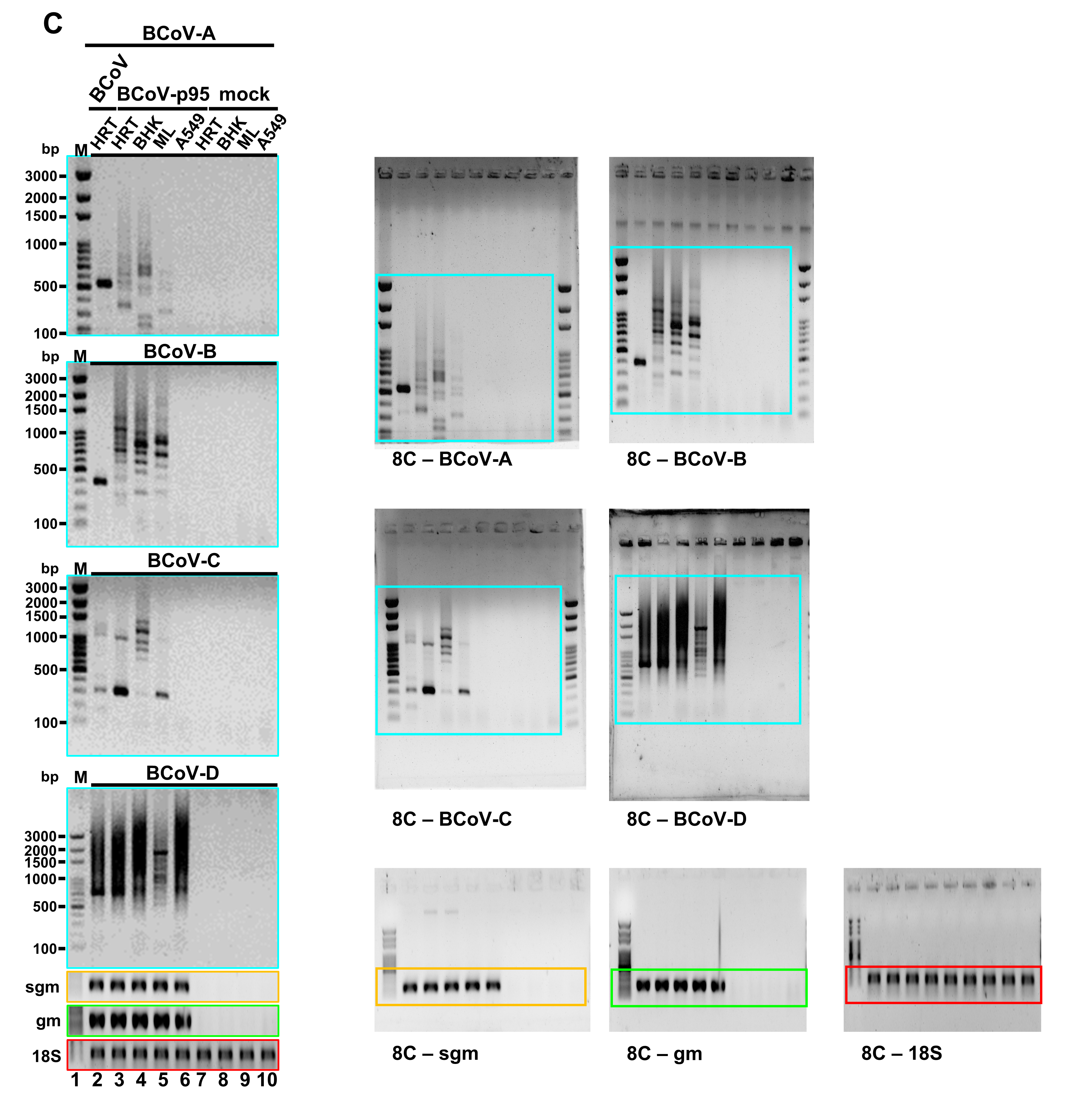


**Figure S6. Uncropped gels for Figure 8C.**


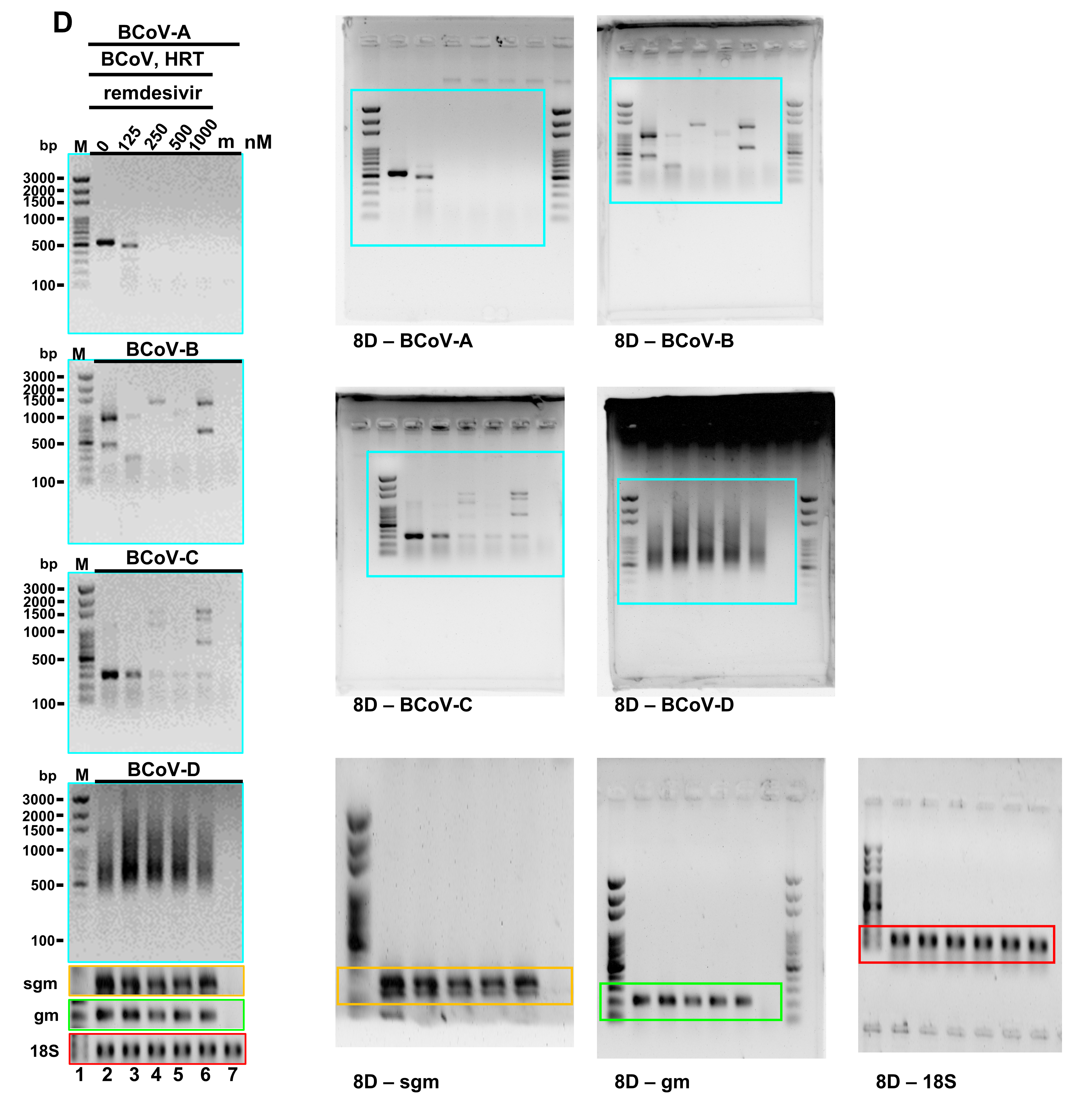


**Figure S7. Uncropped gels for Figure 8D.**


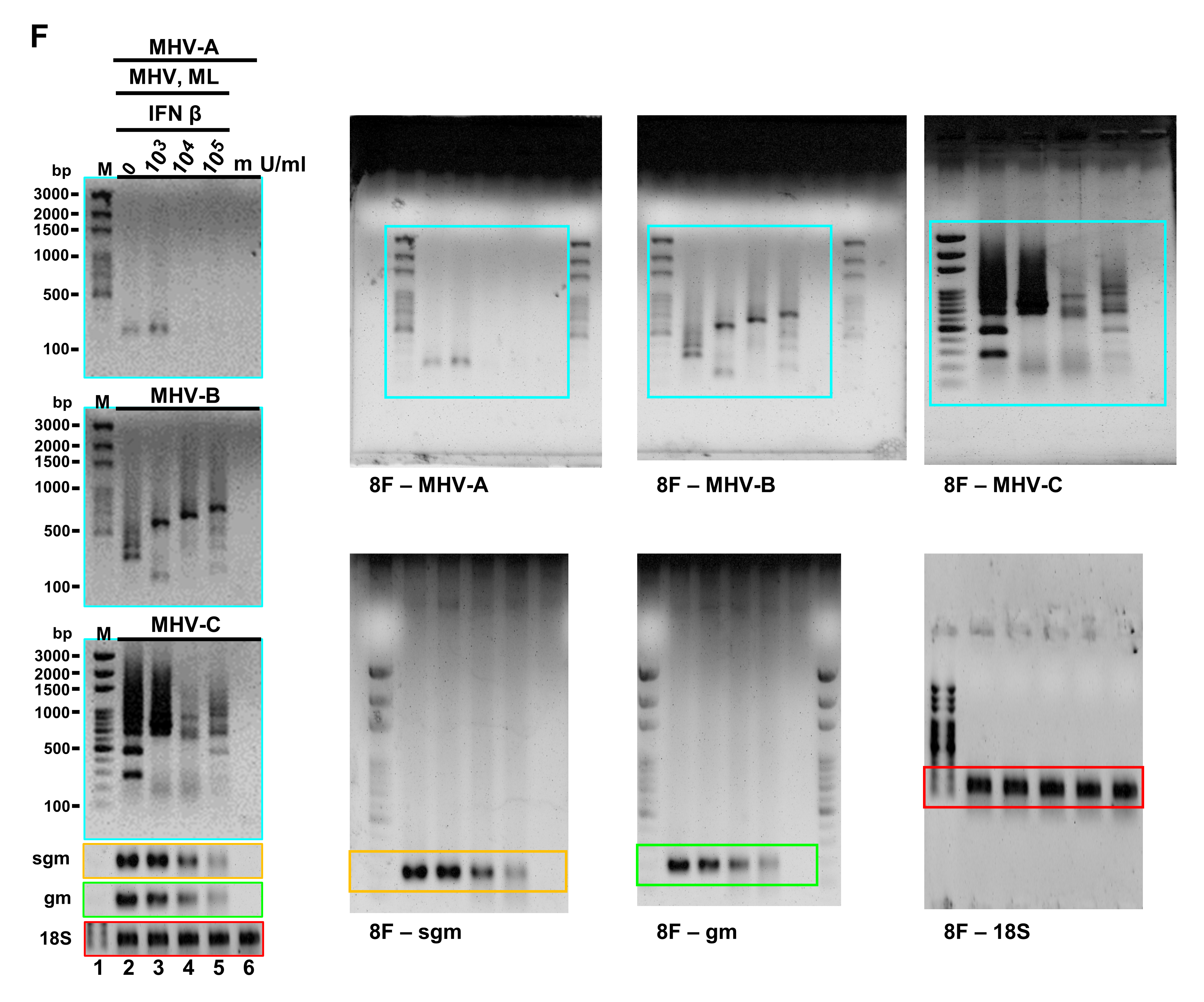


**Figure S8. Uncropped gels for Figure 8F.**

**
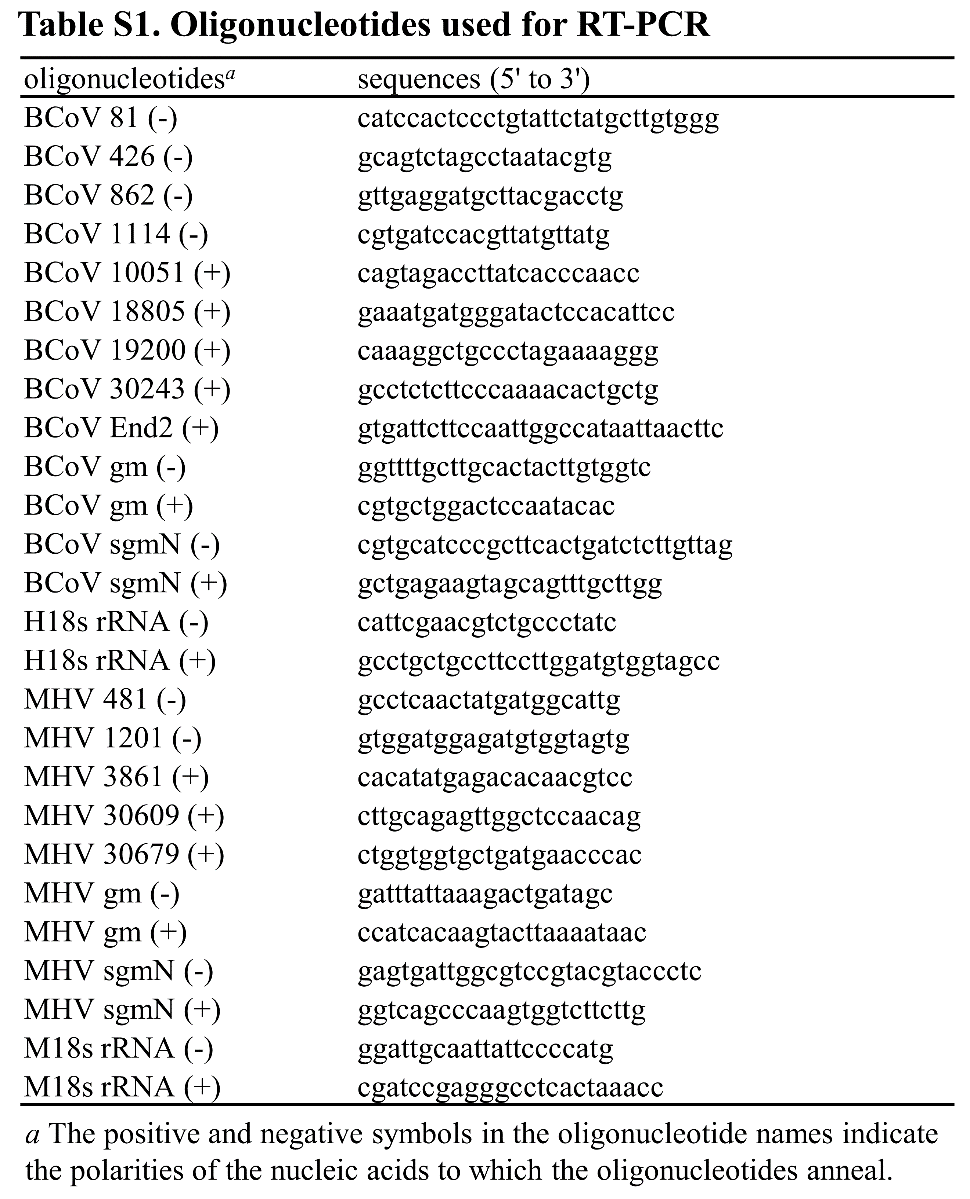
**
